# Physicochemical, Nutritional, and Medicinal Properties of *Opuntia ficus-indica* (L.) Mill. and Its Main Agro-Industrial Use: A Review

**DOI:** 10.3390/plants12071512

**Published:** 2023-03-30

**Authors:** Mariana Martins, Maria H. Ribeiro, Cristina M. M. Almeida

**Affiliations:** 1 Laboratory of Bromatology and Water Quality, Faculty of Pharmacy, Universidade de Lisboa, Av. Prof. Gama Pinto, 2, 1649-003 Lisboa, Portugal; maripmartins9@gmail.com; 2 Research Institute for Medicines (iMed), Faculty of Pharmacy, Universidade de Lisboa, 1649-003 Lisboa, Portugal; mhribeiro@ff.ulisboa.pt

**Keywords:** *Opuntia ficus-indica*, prickly pear, food composition, medicinal properties, agro-industrial applications

## Abstract

The cactus, *Opuntia ficus-indica* (L.) Mill. (OFI) belongs to the *Cactaceae* family, which contains about 130 genera and nearly 1600 species. This review aims to evaluate this plant from several perspectives, namely, botanic, physicochemical, nutritional, and medicinal properties, as well as agro-industrial use. The botanical aspects and morphological characteristics of OFI enable genetic variability, ecological adaptation, and broad geographic distribution. Due to its physicochemical and nutritional composition, it has several medicinal properties appropriate (or suitable) for several industries, such as pharmaceutical, food, and cosmetics. Its fruit, the prickly pear (PP), has potential agro-industrial expansion through the application of different conservation and transformation methods, making it possible to obtain a variety of products. The PP is a source of several nutrients and is an effective system to produce varied foods, which have several advantages from a nutritional, sensory, economic, and shelf-life point of view.

## 1. Introduction

*Opuntia ficus-indica* (L.) Mill. (OFI) belongs to the *Cactaceae* family, characterised by genetic variability, ecological adaptation, and broad geographic distribution (tropical and subtropical regions) [1,2,3]. This plant is originally from South America, namely Mexico, and can be found in the Middle East, South Africa, India, Australia, and some Mediterranean countries [1,2,3]. OFI adapts to extreme weather conditions, has rapid growth in poor soils, and has little need for water [4]. Thus, this species is the most cultivated in the world.

Before starting an OFI plantation, there are several aspects to consider, namely the climate of the place of cultivation, the physical and chemical characteristics of the soil and its preparation, the choice of the cultivar, the planting distances, the orientation crop rows, and the irrigation system [5].

The best harvest time depends on several factors, such as the size, weight, and firmness of the fruit, changes in peel colour, degree of receptacle depth of the flower, the total soluble solids (TSS) or sugar content, the minimum of 14 °Brix, the drop of glochids, the thickness of the peel, the ease of skin removal, and the pulp/peel ratio [6].

OFI consists of leaves, flowers, fruits (prickly pear), cladodes, and roots. OFI presents an ecological adaptation because of the photosynthetic metabolism in cactus cladodes, the crassulacean acid metabolism (CAM). The leaves are only visible on the tender cladodes. They are cylindrical and deciduous, and remain on the plant for over a month [7]. The flowers are large, showy, hermaphroditic, and pollinated by insects or wind; they have no aroma, but they have beautiful colours [4,7]. The prickly pear (PP) is a fleshy, oval, or cylindrical berry 5 cm to 10 cm long and 4 cm to 8 cm wide, and weighs between 80 g and 200 g. The PP shows three components: peel (30 to 40%), pulp (60 to 70%), and seeds (2 to 10%) [7]. The edible part of the fruit has excellent organoleptic characteristics, such as a very aromatic pulp and hard integument seeds, which are excellent mineral sources [4,8].

The diversity of physicochemical and nutritional characteristics of OFI depends on the type of plant (genetic factors), its origin (climate) and agronomic factors (type of cultivation, fertilisation, and irrigation) [5]. The main constituent of cladodes is water, followed by carbohydrates, fibres, minerals (potassium, calcium, magnesium, phosphorus, sodium, manganese, iron, and zinc), proteins, reducing sugars, and lipids [2,9,10,11]. In addition, young cladodes contain ascorbic acid, carotenoids, and chlorophyll [5,12,13]. OFI flower extracts are a source of bioactive substances and have potential use as a food preservative [14]. The flowers predominantly accumulate yellow and red betalains and colourless phenolic compounds (gallic acid, 6-isorhamnetin 3-O-robinobioside, and 7-isorhamnetin 3-O-galactoside) [9,13,15]. The PP comprises water, sugars, ascorbic acid, fibres, amino acids, minerals, and antioxidant compounds such as phenols, flavonoids, betalains, and carotenoids [1,5,13,16]. The energetic value of this fruit varies between 31 and 50 kcal/100 g, comparable to other fruits such as apples, oranges, peaches, and pears [6].

OFI has several properties that allow its use in various applications, such as human and animal feed, in the pharmaceutical, cosmetic and food industries, in civil construction, in alternative fuels, in controlling soil erosion, protecting fauna, and as a source of nectar for bees [2,5,8,17,18].

Regarding the medicinal properties of OFI, several studies confirm that the fruits and cladodes can be used as a source of nutrients and phytochemicals. In this way, OFI is valued for contributing to a healthy diet and because it is rich in health-promoting substances [5,6]. Some medicinal properties of OFI include antioxidant, analgesic, anti-inflammatory, diuretic, anti-diabetic, anti-hypercholesterolaemic and anti-carcinogenic [3,4,5,9,19,20,21,22,23,24,25,26,27]. These properties allow the prevention of hangovers, lipid oxidation–reduction, prevention of some types of cancer, and reduction of the risk of diabetes (type 2) and cardiovascular diseases, two of the most common causes of death worldwide [5,28,29].

During storage, the PP can be contaminated or suffer changes in its physical, chemical, and biological characteristics, leading to losses of some food constituents. Among the most identified alterations are microbial contamination, enzymatic browning, reduced firmness, development of off-flavours, and physical deformations [8]. Furthermore, the PP has a short shelf life of about 3 to 4 weeks, is a low acidic fruit (0.05 to 0.18% citric acid equivalent) and has a pH value of 5.3 to 7.1, which compromises prolonged storage and distribution [9]. Thus, it becomes advantageous to apply conservation and transformation processes to PP: drying, freezing, concentration (physical methods), the addition of sugars, acidification, use of preservatives (chemical methods), and lactic or alcoholic fermentations (biochemical processes) [6].

Some of the products obtained from the various processing methods mentioned above are fresh preserved PP, minimally processed PP, dehydrated PP (dry or osmotically dehydrated), preserves, juices, fermented drinks, liquid sweetener, pulps (frozen or dehydrated), gums or gels, candies, jams or jellies, flours, seed oil, natural dyes, dietary fibres, and thickeners [7,8,30,31].

This review aims to approach the botanical aspects and morphological characteristics of OFI, its cultivation systems, worldwide distribution, physicochemical and nutritional composition, medicinal properties, applications, and agro-industrial uses, namely the conservation and transformation methods applied to the PP.

## 2. *Opuntia ficus-indica* (L.) Mill Species

### 2.1. Botanical Aspects

Cactus refers to the botanical family *Cactaceae*, which encompasses about 1600 species divided into 130 genera, divided into the three subfamilies *Pereskioideae*, *Opuntioideae* and *Cactoideae* [3,9]. In the order *Caryophyllales*, the botanical genus *Opuntia* is the most common, grouping more than 300 species, namely the *Opuntia ficus-indica* (L.) Mill. (OFI) species [1]. The scientific name of this species was attributed in 1700 by the French botanist Joseph Pitton de Tournefort, due to the similarity to thorny plants that grew in the ancient Greek city of Opus. The OFI has various designations, depending on the country and the region where it is located. In Portugal, the common names of this species are prickly pear, agave, devil’s fig tree, and tabaio or tabaibo (Madeira Archipelago) [7].

As a result of the long domestication to which it has been subjected, the OFI is characterised due to its genetic variability. Its taxonomy is complex, and its phenotypes vary according to the environmental conditions it is subjected to, increasing the diversity of adaptive responses. This plant has polyploidy, reproduction occurs sexually and asexually, and several hybrids are interspecific [5]. Thus, the taxonomic classification of this plant becomes difficult, and a detailed study is needed to recognise and identify each species, the varieties, and the adaptations reflected in its phenotype [6]. Table 1 describes the taxonomic classification of the OFI, according to Britton et al. (1919) and Bravo-Hollis et al. (1978) [32,33,34].

OFI is derived from a diploid Mexican ancestry, but polyploidy is favoured by natural hybridisation [35]. Several studies have reported this species as octoploid, heptaploid, pentaploid, hexaploid, and diploid [36,37,38,39]. There is a variation in the number of chromosomes of this species, depending on the origin of the plant [5].

*Cactaceae* have a set of adaptive, evolutionary, and ecological strategies, which give them a great capacity for development in different habitats [8]. OFI adapts to extreme weather conditions and grows rapidly in poor soils with little need for water [4]. Thus, this species is the most cultivated in the world and the most economically important within the genus *Opuntia*, being a viable option in tropical and subtropical regions, where other plants cannot survive, mainly in arid areas (annual rainfall of less than 250 mm) and semi-arid regions (annual rainfall of 250 to 450 mm) [10].

### 2.2. Morphological Characteristics

OFI presents ecological adaptations due to the photosynthetic metabolism that occurs in cacti, namely, crassulacean acid metabolism (CAM). In these plants, the stomata open at night to fix carbon dioxide (CO_2_) and accumulate oxygen (O_2_), simultaneously losing water (H_2_O), which leads to the gradual acidification of the stem. During the day, they keep the stomata closed and prevent the loss of H_2_O through transpiration. The CO_2_ is fixed overnight as malic acid (C_4_H_6_O_5_), which is stored in vacuoles and used during the day [7]. In conditions of extreme water deficiency, stomata remain closed day and night, preventing transpiration and the entry of CO_2_ [6]. In this way, CAM plants increase the efficiency of using H_2_O and the ability to survive in arid and semi-arid environments [5].

**Figure 1:**
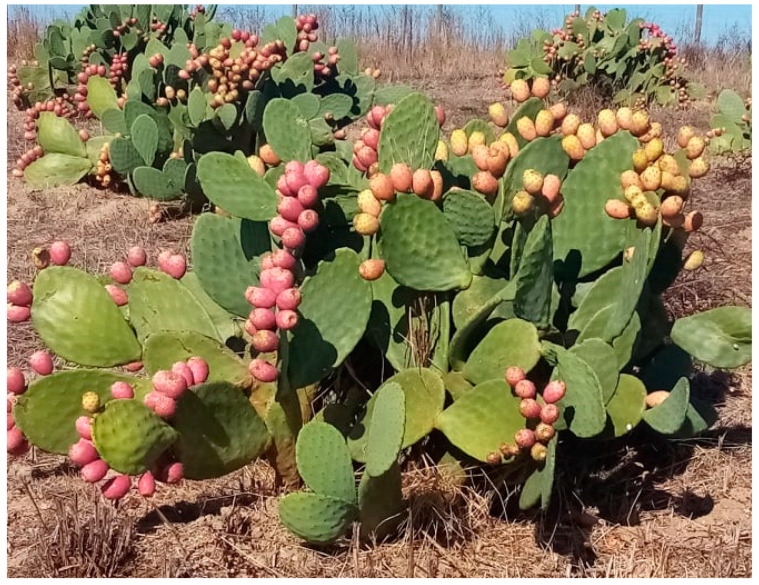
*Opuntia ficus-indica* (L.) Mill.

Anatomically, the OFI consists of roots, cladodes, leaves, flowers, and fruits (Figure 1). OFI can reach up to 5 m in height and is very extensive, fleshy, and densely branched, with fine, absorbent surface roots. The root system develops horizontally, reaching laterally up to 10 to 15 m from the base of the plant [40,41]. The length of the roots is related to the environmental conditions, the type of soil, the availability of water, and the cultivation practices, mainly irrigation and fertilisation [6].

OFI stems, called cladodes, are fleshy and succulent, and are responsible for CAM (Figure 1). They have an ovoid or elongated shape, and their weight ranges from 40 to 100 g. The cladode length varies between 30 and 50 cm, the width varies between 20 and 30 cm, and the thickness varies between 2 and 4 cm. The outer part of the cladode is the chlorenchyma (green part), which is essential for its photosynthetic action, and the inner part of the cladode is the parenchyma, which corresponds to about 50 to 70% of the cladode, where H_2_O and organic acids are stored [9,40,41]. Depending on environmental conditions, the cladodes have areolas, capable of developing new cladodes, flowers, or roots. Areolas have two types of spines in their cavity, serving as a means of defence against damage from prolonged exposure to sunlight and the attack of animals. Large thorns are modified leaves, and the glochids are sharp and grouped in large numbers [6,41]. As the plant ages, the base cladodes lignify and form a trunk-like structure [40].

The leaves develop on the areolas, only visible on the tender cladodes. The leaves are cylindrical and deciduous, remain on the plant for just over a month, and are widely used as animal feed [7,41].

The flowers are large and showy, develop in the upper margin of leaves, are hermaphroditic, and are pollinated by insects or wind [7]. They have no aroma, but beautiful colours, such as yellow, orange, pink, purple, red, or white. Due to the limited flowering duration (March to June), few studies have been carried out on this plant’s flowers [4,41].

**Figure 2:**
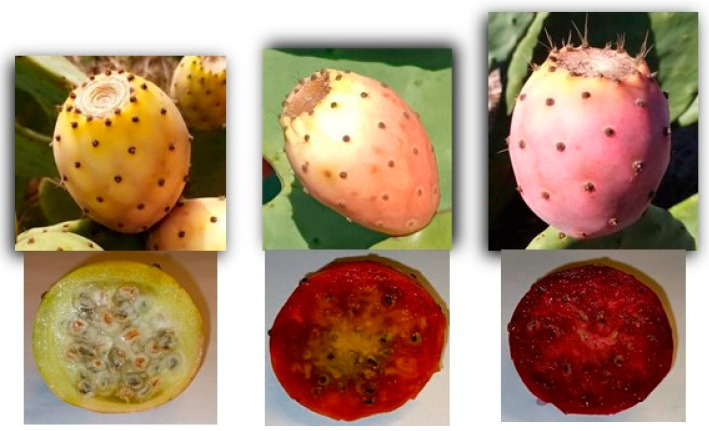
White, orange, and red prickly pear ecotypes and image of the respective cross-sections of the fruits.

The prickly pear (PP) is the OFI fruit that is formed from an inferior ovary located in the stem tissues (Figure 2). Its maturation is completed about 110 to 120 days after flowering, and its final weight can vary between 80 and 200 g. PPs are ovoid or cylindrical, 5 to 10 cm long and 4 to 8 cm wide [7,40].

The PP can be divided into peel, pulp, and seeds. Of the total weight of PP, 30 to 40% corresponds to the peel, 60 to 70% corresponds to the pulp, and 2 to 10% corresponds to the seeds [5,42].

The PP peel can be divided into pericarp and mesocarp. The pericarp is thin and presents the same morphology as the cladodes, including the glochids, and the mesocarp is edible and has nutritional value. However, the mesocarp is not customarily consumed because it is discarded when the fruit is peeled [3,7]. In the initial phase of fruit development, the peel is green, evolving with its maturation to other colours. Depending on the ecotype and variety of the plant, the peel may be greenish-white, yellow, orange, red, purple, or purplish [3,6]. The pulp is the edible portion of the fruit. It is soft, juicy, translucent, gelatinous, and velvety, with a sweet taste. Its colour corresponds to the colour of the peel and it has numerous tiny seeds with hard integuments [3,8]. The seeds, distributed regularly throughout the fruit, are dark, edible and have been extensively investigated [3,43]. The PP’s short shelf life is 3 to 4 weeks, limiting its long-term storage and worldwide distribution [9].

### 2.3. Cultivation System

A successful PP crop must follow specific rules and procedures, always respecting good agricultural practices [44]. The main climatic factors affecting any culture’s yield and quality are temperature, precipitation, sun exposure and wind. Therefore, before starting an OFI plantation, there are several aspects to consider, namely the climate of the place of cultivation, the physical and chemical characteristics of the soil, the choice of the cultivar, the preparation of the soil, the planting distances, the orientation crop rows, and the irrigation system, among other factors [5].

Regarding planting material, this can be obtained from simple asexual vegetative reproduction from whole cladodes or parts of cladodes or through in vitro micropropagation [6]. This last method provides high propagation rates, requires little space for cultivation, allows the production of healthy plants without the intervention of pathogens, selects specific genotypes, avoids physiological disturbances and morphological anomalies, and obtains plants with the desired characteristics [45]. Khalafalla et al. (2007) demonstrated that OFI micropropagation, through areolas, can combat desertification in arid and semi-arid areas [46].

The PP is a perennial crop with an annual cycle, which can occur naturally with a second flowering and subsequent fruiting when favourable conditions exist [40]. The physiological development of the PP occurs between 70 and 150 days after flowering, depending on the production season, variety/ecotype, climatic conditions, and post-harvest treatments. This development begins with an intense cell multiplication, followed by an increase in volume, which gives rise to the appearance of vacuoles, which accumulate nutrients [8]. There are three phases to fruit growth: dry matter growth of the husk, the development of dry matter of the seeds, and the development of the pulp. During maturation, changes occur in the colour of the peel and the texture of the fruit, the drop of glochids occurs, and the total soluble solids (TSS) content increases [40].

The parameters that help define the best PP harvest time are size, weight, and firmness of the fruit, changes in peel colour, degree of receptacle depth of the flower, the SST content, the minimum of 14 °Brix, the drop of glochids, the thickness of the peel, the ease of skin removal, and the pulp/peel ratio [6].

PPs are difficult to harvest due to glochids and spines, which can pierce the peel and enter the eyes and respiratory system of the person performing the collection. Therefore, this is carried out manually, during the night and until the beginning of the morning, when there is greater ease of cutting, better resistance to damage, and higher maintenance of turgor of the fruit tissues, in which the glochids are more humid and attached to the fruit [5]. There are several harvesting techniques: turning or twisting, which is a procedure that causes damage to the fruit and is used when the product is intended for processing; cutting flush to the insert, through which fruits are obtained with little conservation time, used when the fruit is for immediate consumption or sale; and cutting a small piece of the cladode attached to the fruit, which is the most used technique for the commercialisation of PP, as it allows an increase in the time of conservation [40].

After harvesting, the glochids are removed mechanically without damaging the epidermis of the fruits. Then the fruits are selected according to the purpose for which they are envisioned (or “for the purposed application”). Subsequently, the fruits are washed with drinking water or chlorinated with sodium hypochlorite to reduce the microbial load. Then the fruits are waxed by immersion in, or sprinkling of, wax, to control the loss of water by transpiration, reduce the intensity of the fruits’ gas exchange, improve the visual appearance, and prolong their conservation. The classification of PPs is performed manually or mechanically, according to colour and size [7,8]. Generally, the fruits are packaged on the day of harvest and transported to their destination in refrigerated conditions. If the fruits are handled a few days after harvest, they must be stored under certain conditions. For PP storage, low temperatures are recommended, between 5 °C and 8 °C, with a relative humidity between 85% and 95%, depending on various factors such as storage time, type of packaging, the season of harvest, and the variety of the fruit [8]. Under these conditions, the shelf life of the fruits varies between 3 and 8 weeks [40]. The *Codex Alimentarius* contains standards that describe the quality, presentation, packaging, and hygiene requirements relating to the PP [47].

Like other cultures, OFI can also suffer biotic diseases (fungal, bacterial, and viral infections) and abiotic diseases from frost, hail, herbicides, pesticides, and fruit splitting [5]. In Portugal, the PP species are relatively resistant to pests and diseases. Due to the resistance of this plant and the climatic conditions, which are not unfavourable, there are no stressful situations. Concerning diseases, these are mainly caused by fungi or bacteria and pests. The Mediterranean fly (*Ceratitis capitata*) is the insect that causes the most damage in the culture of PP, but damage can also be caused by ants, slugs, snails, mealybugs, and fruit flies [7,44].

### 2.4. Worldwide Distribution

The OFI originates from South America, namely Mexico. This species was introduced in Spain, by Christopher Columbus, in 1493, on one of his trips to America. Subsequently, the OFI was dispersed and naturalised in the Mediterranean area of Europe and northern Africa [35,41].

Due to its genetic variability, OFI has a high adaptability, ecological and, consequently, a wide geographic distribution, can be found in locations with diverse climatic conditions, including North, Central and South America, Northern, Central and Southern Africa, Middle East, Australia, India, and some Mediterranean countries [2,3].

The leading producers and consumers of OFI are Mexico and Italy, and it is in Mexico that this species has the highest degree of genetic diversity. Of the approximately 590,000 ha grown worldwide, Mexico and Italy contain 70% and 3.3%, respectively [10,29]. In Mexico, cladodes are the fifth most consumed vegetable, and PP is the third most consumed fruit [37].

In Europe, the economic and agricultural importance of the PP dates to the 16th century. The OFI began by decorating bourgeois-class gardens and properties, and then served as a hedge to delimit rural properties due to the thorns and, later, its fruits became a food source for the lower classes in times of food scarcity. Currently, PP presents itself in different forms, from fresh to processed, also being sold as a gourmet product [7,41].

In Portugal, mainly in the Alentejo and Algarve, the cultivation of OFI is allowed, being an introduced, naturalised, non-invasive species, which is in the phase of expansion, often being found on the edges of rural roads and paths or even to delimit private land [42,44]. Recently, the private sector has started focusing on this species’ growth for fruit production in the semi-arid areas of the Alentejo and Algarve. Since 2009, with the help of a programme for unemployed young farmers, more than 200 ha have been planted, and a further 500 ha will be planted in the coming years [5].

## 3. Physicochemical and Nutritional Composition

### 3.1. Cladodes and Flowers

The physicochemical composition of OFI depends on the plant part, ecotype or variety, environmental factors, growing area, type of fertilisation, season, the state of maturation, and post-harvest procedures. Therefore, the nutritional values vary between species, varieties, and plant parts, and should not be taken as absolute values [3,5].

The cladodes are fleshy and adapt to arid environments, their primary function being water storage [7]. For this reason, the main constituent of cladodes is water (88–95%), and, consequently, cladodes are a low-calorie food (27 kcal/100 g) [2]. In their chemical composition, based on fresh weight, the water content is followed by carbohydrates (3–7%), fibre (1–2%), minerals (1–2%), proteins (0.5–1%), reducing sugars (0.64–0.88%), and lipids (0.2%) [2,9,10,11]. In addition to these constituents, young cladodes contain ascorbic acid (10–15 mg/100 g), carotenoids (30 μg/100 g), mainly β-carotene, and chlorophyll (12.5 mg/100 g) [5,12].

Regarding minerals, cladodes contain potassium (2.35–55.20 mg/100 g), calcium (5.64–17.95 mg/100 g), magnesium (8.8 mg/100 g), phosphorus (0.15–2.59 mg/100 g), sodium (0.3–0.4 mg/100 g), manganese (0.19–0.29 mg/100 g), iron (0.09 mg/100 g), and zinc (0.08 mg/100 g) [1,48].

Several studies with cladode juices have shown pH values of around 4.6, with 0.45% titratable acids (low-acid vegetable) and 6.9% total soluble solids (TSS) [11,15]. The cladodes are characterised by fluctuating levels of malic acid (95–985 mg/100 g) due to CAM. During the night, OFI fixes CO_2_ as C_4_H_6_O_5_, releasing O_2_, and during the day C_4_H_6_O_5_ is decarboxylated, and the released CO_2_ is converted into glucose (C_6_H_12_O_6_) through photosynthetic action [49]. Other organic acids are also present in cladodes, including citric acid (31–178 mg/100 g) [50], and oxalic acid, which occurs in a dissolved (0.61 mg/g dry weight) or crystalline form (34.5 mg/g dry weight) when calcium sequestration occurs, forming calcium oxalate crystals (11.5–14.3 mg/100 g) [1,51].

One of the main constituents of cladodes is dietary fibre, a class of compounds characterised by a mixture of polymeric carbohydrates of vegetable nature, and oligosaccharides or polysaccharides, such as cellulose, hemicellulose, pectic substances or resistant starch, which may be associated with lignin and other components such as polyphenols, waxes, saponins, cutin, phytates, or proteins. The benefits associated with dietary fibre intake include the decreased risk of coronary heart disease, diabetes, obesity, and cancer [6]. Total dietary fibre (TDF) is classified according to its solubility in water in two fractions: soluble dietary fibre (SDF) and insoluble dietary fibre (IDF) [52]. The SDF is composed of mucilage, gums, pectins, and some hemicelluloses, and is associated with the reduction of cholesterol levels and the control of the absorption of glucose. IDF is composed of cellulose, lignin, and most hemicelluloses, and presents water-binding capacity, which increases stool weight, ion exchange, and absorption of bile acids, minerals, and vitamins, as well as interactions with the microbial flora [6]. Regarding IDF, cladodes contain cellulose (11%), hemicellulose (8%), and lignin (3.9%) [2].

Mucilage is a complex polysaccharide produced by specialised cells of the cell wall, and forms molecular networks capable of retaining large amounts of water [17]. In cladodes, mucilage is composed of arabinose (42%), xylose (22%), galactose (21%), galacturonic acid (8%), and rhamnose (7%) [53].

The age of cladodes, the environmental conditions, the soil type, and the climate may explain variations in the polyphenol content of cladodes: nicotiflorin (2.89–146.5 mg/100 g), narcissine (14.69–137.1 mg/100 g), isoquercetin (2.29–39.67 mg/100 g), ferulic acid (0.56–34.77 mg/100 g), isorhamnetin-3-*O*-glucoside (4.59–32.21 mg/100 g), rutin (2.36–26.17 mg/100 g), coumaric acid (14.08–16.18 mg/100 g), 3,4-dihydroxybenzoic acid (0.06–5.02 mg/100 g), 4-hydroxybenzoic acid (0.5–4.72 mg/100 g), salicylic acid (0.58–3.54 mg/100 g) and gallic acid (0.64–2.37 mg/100 g) [1,13,54]. The carbohydrates, carotenoids, and acidity increase during development, and the proteins and fibres decrease with cladode age [11].

Several studies show a great interest in OFI flower extracts, e.g., as a source of bioactive substances and potential use as a food preservative [14]. The flowers accumulate predominantly yellow and red betalains (betaxanthins and betacyanins, respectively), and colourless phenolic compounds [3,9,48]. Regarding the phenolic content, the main compounds present in the flowers are gallic acid (1630–4900 mg/100 g), 6-isorhamnetin 3-O-robinobioside (4269 mg/100 g), and 7-isorhamnetin 3-*O*-galactoside (979 mg/100 g) [1,3,55].

Bousbia et al. (2022) did a study to evaluate the phytochemicals and antioxidant activity of the peels, flowers, and seeds of OFI from Souk-Ahras, Algeria. They determined the content of total polyphenols, flavonoids, and betalains, and the antioxidant activities by DPPH, FRAP (ferric reducing antioxidant power) and TAC (total antioxidant capacity) tests, and concluded that flowers were the organ richest in polyphenols, and the flavonoids and peel had a high content of betalains. The flowers and seeds presented considerable DPPH free radical scavenging power, and the peel extracts showed an important total antioxidant capacity and a consequent ferric iron reducing power [56].

A study by Ammar et al. (2012) on the composition of OFI flowers during and after flowering indicates that the main compounds present in hexane extracts are carboxylic acid (28–97%), terpenes (0.2–57%), esters (0.2–27%), and alcohols (<1.8%). This study also demonstrates antibacterial activity against *Pseudomonas aeruginosa*, *Staphylococcus aureus*, and *Escherichia coli*, and antifungal activity against *Aspergillus niger* and *Candida lipolytica*. It should be noted that the post-flowering phase corresponds to the accumulation maximum of phenolic compounds, and, consequently, there is an increase in the antioxidant and antibacterial properties [14]. Regarding the composition of vitamins in the OFI’s flowers, the results are not very conclusive, given the small number of studies [1,3].

### 3.2. Prickly Pears

Due to the wide varieties/ecotypes of OFI and its wide geographic distribution, information on the physicochemical and nutritional composition of the PP is scarce and varied [57]. The type of plant (genetic factors), the origin of the plant (climate) and the agronomic characteristics (type of cultivation, fertilisation, and irrigation) are decisive in the diversity of physicochemical and nutritional parameters of the fruit [5,6]. Table 2, Table 3 and Table 4 [58,59,60] compile various data regarding the physical–chemical composition and nutritional status of various PP ecotypes in different regions.

Martins et al. (2022) completed a study on three PP ecotypes (white, orange, and red) cultivated in Portugal, mainly in the south of the country, in the Alentejo and Algarve. This team characterised these PPs according to the morphometric, physicochemical, nutritional, and toxic profiles of the fruit and mesocarp. The three ecotypes showed similar physicochemical and nutritional profiles: 79.9 to 83.0% of water content in fruits and 82.6 to 84.8% of water content in mesocarps; 14 to 20% of total carbohydrate contents, with 8 to 14% corresponding to reducing sugars; 1.4% total dietary fibre in fruits and 4.8% total dietary fibre in mesocarps; protein values in the order of 0.5%; fat values between 0.12% and 0.15%; 21 to 32 mg/100 g of ascorbic acid; 18 to 43 mg/100 g of carotenoids; and 109 to 158 mg/100 g of total polyphenols. All ecotypes had a low caloric content, varying between 150.0 and 160.7 kcal/100 g. This study also included the analysis of 30 minerals (essential nutritive, non-nutritive, and toxic macrominerals and microminerals) of the PP fruits and mesocarps. The macromineral with the highest concentration was potassium (140–650 mg/100 g), followed by calcium (18–286 mg/100 g), magnesium (13–96 mg/100 g), phosphorus (3.7–25 mg/100 g), and sodium (1.9–4.3 mg/100 g). The majority of nutritive microminerals were silicon (362–3993 µg/100 g), manganese (714–5866 µg/100 g), zinc (151–301 µg/100 g), nickel (72–128 µg/100 g), copper (67–128 µg/100 g), and iron (72–126 µg/100 g). Of the non-nutritive microminerals, the ones that were most evident in the three were aluminium (301–2459 µg/100 g), strontium (50–504 µg/100 g), and boron (95–198 µg/100 g). The toxic microminerals that stood out were barium (150–676 µg/100 g) and lithium (91–257 µg/100 g) [42].

Stintzing et al. (2003) studied several properties and characteristics of three Italian PP ecotypes: red, orange–yellow and white–green. The densities for each ecotype were 1.0543 g/cm^3^, 1.0479 g/cm^3^ and 1.0535 g/cm^3^, respectively. The pH ranged from 5.7 to 6.3, the content of TSS ranged from 12.3 to 13.7 °Brix, and the acidity content ranged from 0.05% to 0.11% [61].

**Table 2:** Physicochemical and nutritional composition of several PP ecotypes in different regions of the world.

Country	Ecotype	DM (%)	Water (%)	Ashes (%)	Carb (%)	Prot (%)	Lip (%)	DF (%)	AA (mg/100 g)	Carot (µg/100 g)	PC (mg/100 g)	Ref.
Mexico	Values for 18 wild and cultivated varieties of Potosino-Zacatecano	10–15	85–90	---	T: 10–17 RS: 5–14	1.4–1.6	0.50	2.40	4.6–41	TE	---	[58]
	*Naranjona* (yellow/orange)	---	---	---	---	---	---	---	1.5 –2.0	50–100	10–65	[59]
	*Pelon rojo* (red)	7.52	92.48	13.14	---	TE	0.94	SDF: 8.12 IDF: 19.39	---	$1.5\times 10^{6}$	1.54	[27]
		---	---	---	---	---	---	---	1–1.5	<50	50–95	[59]
	*Alfajayucan* (green)	5.41	94.59	17.07	---	TE	1.40	SDF: 7.98 IDF: 34.95	---	---	2760	[27]
Italy (Sicily and Apulia)	White	---	---	---	Glu: 6.4 Fru: 5.7	0.33	---	---	33	---	---	[60]
	Red/purple	---	---	---	Glu: 6.0 Fru: 5.4	0.33	---	---	31	---	---	
		---	---	---	Glu: 5.6 Fru: 4.3	---	---	---	22	---	---	[61]
		16.70	83.30	---	Glu: 1.88 Fru: 0.78	---	---	---	36.6	---	89.2	[62]
	Yellow/orange	---	---	---	Glu: 6.2 Fru: 6.0	0.34	---	---	38	---	---	[60]
		---	---	---	---	---	---	---	26.9	---	0.746	[26]
		---	---	---	---	---	---	---	29	1.5	---	[63]
		16.10	83.90	---	Glu: 2.14 Fru: 1.04	---	---	---	30.2	---	69.8	[62]
Spain	Green	17.99	82.01	0.41	---	0.87	0.48	5.65	17.1	---	45.0	[64]
	Orange	17.39	82.61	0.37	---	0.94	0.53	4.86	17.2	---	45.4	
	Red	---	---	---	---	---	---	---	18.5	2.58	218.8	[65]
USA (Texas and California)	Green	---	---	---	---	---	---	---	45.8	290	---	[66]
		---	---	---	---	---	---	---	51.1	---	242	[2]
	Orange	---	---	---	---	---	---	---	70.2	---	247	
	Red	---	---	---	---	---	---	---	67.9	---	335	
	Purple	---	---	---	---	---	---	---	95.4	---	660	
Argentina	---	15.80	84.20	0.51	10.27	0.99	0.24	3.16	22.56	---	---	[6]
	Yellow	14–18	82–86	---	---	---	---	---	25–32	---	60–77	[67]
Chile	---	16.20	83.80	0.44	14.06	0.82	0.09	0.23	20.33	530	---	[6]
	Orange	---	---	---	---	---	---	---	---	984	371.95	[68]
	Purple	---	---	---	---	---	---	---	---	199.9	777.43	
North Africa (Morocco and Algeria)	Morocco–Yellow	14.49	85.51	0.29	RS: 15.23	0.28	---	---	---	---	---	[69]
	---	---	---	---	---	---	---	---	---	---	1.50	[25]
	Morocco–Red	---	---	---	---	---	---	---	---	---	1.78	
	Algeria	5.60	94.40	1.0	Glu: 29 Fru: 24 Sac: 0.19	1.45	0.70	---	---	---	---	[70]
Egypt	Average values of six local varieties	17.72–19.24	80.76–82.28	1.85–2.12	Suc: 14.9	5.09–7.62	2.52–3.17	---	1050–1200	---	---	[71]
	---	14.90	85.10	0.40	---	0.80	0.70	0.10	25	---	---	[72]
Portugal	White (only fruit)	17.4	82.6	0.43	T: 14.3 RS: 10.7	0.49	0.15	TDF: 1.5	26.5	42800	37.3	[42]
	Orange (only fruit)	15.5	84.5	0.57	T: 18.7 RS: 14.2	0.56	0.12	TDF: 1.7	21.4	25600	43.9	
	Red (only fruit)	15.2	84.8	0.49	T: 17.5 RS: 12.9	0.50	0.10	TDF: 1.2	20.6	18400	46.2	
South Africa (Cape)	Five different cultivars	15.3–19.2	80.8–84.7	---	T: 8.26–11.4 Glu: 4.44–6.18 Fru: 3.25–5.24 Suc: 0.08–0.24	---	---	---	---	---	---	[19]
Saudi Arabia	Green to brownish-orange	14.40	85.60	0.44	12.8 (60:40 Glu/Fru)	0.21	0.12	0.02	22	TE	---	[73]
South Korea	---	9.30	90.70	12.12	Suc: 68.7 Fru: 18 Glu: 12.8 Man: 0.5	4.24 (extract free of N = 69.2%)	1.35	3.79	163.8	---	497.6	[74]

Legend: DM = dry matter; Carb = carbohydrates; Prot = proteins; Lip = lipids; DF = dietary fibre; AA = ascorbic acid; Carot = carotenoids; PC = phenolic compounds; T = totals; RS = reducing sugars; Glu = glucose; Fru = fructose; Sac = saccharose; Suc = sucrose; Man = mannose; TE = trace elements; TDF: total dietary fibre; SDF = soluble dietary fibre; IDF = insoluble dietary fibre.

**Table 3:** Physicochemical composition of several PP ecotypes in different regions of the world.

Country	Ecotype	pH	Acidity (%)	TSS (°Brix)	Pig (mg/100 g or mg/100 mL)	Ref.
Mexico	Values for 18 wild and cultivated varieties of Potosino-Zacatecano	6.5–7.1	0.021–0.049	11–16	Chlorophyll: 0.2–1.1	[58]
	Naranjona (yellow/orange)	5.7–6.6	0.05–0.09	9–12.5	Xanthophyll: 45–69	[75]
	Pelon rojo (red)	---	---	11.9–14	---	[59]
Italy (Sicily and Apulia)	White	6.4	0.02	---	---	[60]
	Red/purple	6.40	0.02	---	---	
		---	---	---	Indicaxanthin: 2.61 Betanine: 5.12	[76]
		5.70	0.11	13.6	---	[61]
		5.89	---	12.7	Betacyanin: 39.3	[62]
	Yellow/Orange	6.02	---	12.1	Betacyanin: 3.6	
		6.44	0.02	---	---	[60]
		---	---	---	Indicaxanthin: 8.42 Betanine: 1.04	[76]
		5.70	0.06	12.3	---	[61]
		---	---	---	Indicaxanthin: 9.3 Betanine: 1.21	[63]
Spain	Green	6.39	0.072	14.98	---	[64]
	Orange	6.22	---	14.05	---	
		---	---	---	Indicaxanthin: absence Betaxanthin: 25	[77]
	Red	---	---	---	Indicaxanthin: 19 Betaxanthin: 25.4–30 Betacyanin: 15.2	
USA (Texas and California)	Green	6.5	---	14.2	Betaxanthin: 4 Betacyanin: 1	[2]
	Orange	6.3	---	12.6	Betaxanthin: 763 Betacyanin: 66	
	Red	5.6	---	14.8	Betaxanthin: 679 Betacyanin: 1200	
	Purple	6.3	---	12.8	Betaxanthin: 1958 Betacyanin: 431	
Argentina	---	5.95	0.14	15.41	---	[6]
	Yellow	---	---	14–16	---	[67]
	Dark purple	---	---	11.3	Betaxanthin: 14 Betacyanin: 34.4	[78]
	Purple	---	---	15	Betaxanthin: 11.8 Betacyanin: 28	
	Green	---	---	15	Betaxanthin: 0.31 Betacyanin: 0.08	
Chile	---	6.37	0.06	14.1	---	[6]
	---	---	0.078	16.5	---	[79]
	Orange	---	---	---	Indicaxanthin: 8.94 Betaxanthin: 2.93	[68]
	Purple	---	---	---	Indicaxanthin: 0.21 Betaxanthin: 11.1	
North Africa (Morocco)	Yellow	6.22	0.056	14.9	---	[69]
		---	---	---	Indicaxanthin: absence Betaxanthin: 3.78	[25]
	Red	---	---	---	Indicaxanthin: 5.65 Betaxanthin: 4.59	
Egypt	Average values of six local varieties	---	0.24–0.32	12.87–12.94	---	[71]
	---	5.8	0.05	13.2	---	[72]
Portugal	White (only fruit)	5.5	---	16.9	---	[42]
	Orange (only fruit)	5.4	---	19.1	---	
	Red (only fruit)	5.5	---	16.6	---	
South Africa (Cape)	Five different cultivars	6.13–6.38	0.02–0.03	10.2–13.9	---	[19]
Saudi Arabia	Green to brownish-orange	5.75	0.18	14.2	---	[73]

Legend: TSS = total soluble solids; Pig = pigments.

**Table 4:** Mineral composition of several PP ecotypes in different regions of the world.

Minerals	Mexico, Naranjona [80]	Italy, White [61]	Italy, Yellow/Orange [61]	Italy, Red/Purple [61]	Spain, Green [64]	Spain, Orange [64]	Argentina [6]	Chile [6]	Morocco, Yellow [69]	Algeria [70]	Egypt Medium Values, Six Varieties [71]	Egypt [72]	Egypt Medium Values, Five Varieties [6]	Portugal, White [42]	Portugal, Or-Ange [42]	Por-Tugal, Red [42]	Saudi Arabia [73]	South Korea [74]
mg/100 g																		
Cl	3560	---	---	---	---	---	---	---	---	---	---	---	---	---	---	---	---	---
K	610	2321	2558	2831	159.5	156.7	78.72	217	183.9	199	400–510	90	---	140	151	152	161	2609
Mg	100	141.8	143	153	26.7	23.1	---	16.1	---	18.8	40	98.4	18.7–38.6	21	18	13	27.7	800.6
Ca	110	76.2	86.7	88.7	24.4	28.8	---	12.8	21.46	12.4	110–130	24.4	28.3–56.4	18	45	23	27.6	2087
Na	160	10.7	12.4	16.9	0.524	0.758	1.64	0.6	13.3	1.09	90–150	1.1	---	1.9	2.2	3.1	0.8	539.7
P	50	---	---	---	---	---	---	32.8	---	---	100–120	9.22	---	18	16	3.7	14.4	99.6
Mn	33	---	---	---	0.301	0.306	---	---	---	---	---	---	0.133–0.403	1.340	0.820	0.714	---	2.2
Fe	28	---	---	---	0.2	0.195	---	0.4	---	---	---	---	0.303–0.45	0.102	0.072	0.126	1.5	12.9
Zn	16	---	---	---	0.204	0.207	---	---	0.029	---	---	---	0.245–0.732	0.214	0.176	0.151	---	---
Cu	5	---	---	---	0.0384	0.0396	---	---	0.019	---	---	---	0.085–0.195	0.128	0.067	0.094	---	---
Ni	---	---	---	---	0.0289	0.0268	---	---	---	---	---	---	0.033–0.086	0.082	0.076	0.072	---	---
Mo	0.3	---	---	---	---	---	---	---	---	---	---	---	---	0.0039	0.0027	0.0035	---	---
Cr	---	---	---	---	0.0115	0.0102	---	---	---	---	---	---	0.018–0.024	0.0018	0.0034	0.0011	---	---
Co	---	---	---	---	---	---	---	---	---	---	---	---	0.017–0.029	0.0035	0.002	0.0018	---	---
Si	---	---	---	---	---	---	---	---	---	---	---	---	---	0.514	<0.362	3.993	---	---
Al	---	---	---	---	---	---	---	---	---	---	---	---	---	0.370	0.301	2.459	---	---
B	---	---	---	---	---	---	---	---	---	---	---	---	---	0.159	0.095	0.167	---	---
Sr	---	---	---	---	---	---	---	---	---	---	---	---	---	0.050	0.073	0.082	---	---
Ba	---	---	---	---	---	---	---	---	---	---	---	---	---	0.250	0.153	0.150	---	---
Li	---	---	---	---	---	---	---	---	---	---	---	---	---					---

Medina et al. (2007) also carried out a similar study with two ecotypes of the PP (orange and green), from the island of Tenerife, in Spain. The different locations of PP resulted in some differences in their physicochemical composition. The refractive index of the orange ecotype was 1.35 and the refractive index of the green ecotype was 1.36, and for both the average pH value was 6.32, the average TSS content was 14.58 °Brix, and the average acidity content was 0.078% [64].

As previously mentioned, the PP can be divided into three components: peel (30 to 40%), seeds (2 to 10%), and pulp (60 to 70%) [5]. The peel generally contains high levels of cellulose, calcium, and potassium, the seeds contain cellulose and considerable amounts of proteins and lipids, and the pulp is rich in glucose, fructose, and pectin [3,81]. El-Gharras et al. (2006) evaluated the alterations of the physicochemical characteristics of the PP in three maturity stages. The pulp’s pH, sugar, protein, and calcium levels increase as it matures, while the peel, and seeds’ acidity and moisture decrease [69].

In a study concerning the chemical composition of the pulp, peel, and seeds of the fruits cultivated in Algeria, Salim et al. (2009) observed that the pulp and the peel contained a higher water content (94.40 and 90.33%, respectively) than the seeds (18.05%). These had a higher protein content (4.48%) than the pulp and the peel, with 1.45%. The same observation was made for lipids, where the seeds had a more significant amount (3.66%) than the pulp (0.7%) or the peel (1.06%). Regarding the content of ashes, this was higher in the seeds (12.66%) than in the pulp (1%) or in the peel (3.05%). The content of total carbohydrates in the seeds was higher (61.15%) than in the peel (4.11%) and in the pulp (2.45%). The pulp had less sucrose and more glucose and fructose than the peel. The most representative macrominerals in the three components of the fruit were, in descending order, potassium, magnesium, calcium, and sodium [70].

The water content in the pulp is protected by the peel, which is thick and rich in mucilage. This property binds strongly to water, helping to prevent the fruit from drying out [6]. The peel is more acidic than the pulp and has a higher phenolic content. It contains considerable amounts of polyunsaturated fatty acids, particularly linoleic acid, and other fat-soluble compounds such as sterols (mainly β-sitosterol), β-carotene, and vitamin K1 [5,34,82,83].

PP seed oil is rich in unsaturated fatty acids, presenting a high linoleic acid and low linolenic acid content. It also contains tocopherols (mainly ϒ-tocopherol), which prevent lipid peroxidation, making this oil quite stable. Considering the physicochemical properties referred to above, as well as others, namely the refractive index, the iodine index, and the saponification number, PP seed oil has properties like sunflower or grape seed oils [5,48,83].

The PP pulp has a low acidity, with predominately, in descending order, the following organic acids: malic, quinic, shikimic, oxalic, and citric acids (may vary between 0.05 and 0.18%) [83]. In this way, the PP is characterised as a low-acid food with a pH between 5.6 and 6.5. The sugar content of the PP pulp is relatively high, ranging from 12 to 17%. The carbohydrates in this fruit consist of the reducing sugar agents glucose (53%) and fructose (47%), in a 1:1 ratio [5]. Glucose represents an instantly available energy source for the brain, while fructose enhances the flavour of the fruit [9]. Given the high sugar and low acid contents, the pulp has sugar:acid ratios in the range of 90:1 to 490:1 [1]. The levels of reducing sugars in PP pulp are superior to those of other fruits, such as apples, pears, peaches, plums, strawberries and raspberries [69].

The protein content represents only a tiny percentage, between 0.21% and 1.6%, increasing during the PP maturation phases. The amount of this nutrient in the PP is comparable to that found in other fruits such as apples, pears, apricots, pineapples, oranges, and peaches [6,58,69].

The total free amino acid content of PP pulp is higher than most other fruits and similar only to oranges and grapes, with the leading free amino acids being proline, taurine, and serine [13,59,83].

Typically, the fruit pulp contains low levels of lipids; in the case of PP, the pulp is no different. The lipid content varies between 0.1% and 1.0%, representing about 8.70 g of total lipids/kg dry weight [48]. While we can find a significant amount of neutral lipids (87% of total lipids) in the seed oil, the concentration of polar lipid compounds is superior in pulp oil (52.9% of total lipids) [57]. In this oil, the fattiest acid is linoleic acid, followed by palmitic, oleic, and linolenic acids. The main sterols reported in pulp oils are β-sitosterol and campesterol, constituting about 90% of the total sterols [13,83]. In addition, tocopherols (α-, β- and δ-), β-carotene, and phylloquinone (vitamin K1) present in seeds and pulp oils and protect lipids from oxidative damage [57].

In the PP pulp, the TDF content can vary between 0.02% and 3.15%, a percentage significantly higher than for some of the most consumed fruits and vegetables [84]. Pectin is responsible for the PP pulp viscosity, an essential element in producing juices, marmalades, and jellies. However, more studies are needed to fully characterise the PP pulp’s hydrocolloid fraction, composed of arabinose, galactose, rhamnose, and galacturonic acid [83].

The PP pulp has a high level of ascorbic acid, reaching values of 40 mg/100 g, higher than for apples, bananas, grapes, and pears but comparable to oranges, lemons, and papayas [6,7]. PP is also rich in carotenoids, mainly β-carotene (0.53 mg/100 g) [7]. Typically, the highest ascorbic acid content is present in PP with red peel and the highest content of carotenoids is present in PP with yellow peel [10]. The vitamins B, E, and K are present in the PP pulp in trace concentrations. As mentioned, carotenoids, tocopherols, and vitamin K1 play an important role in lipid protection due to their antioxidant properties [57].

Kuti et al. (2004) investigated the antioxidant compounds present in extracts of four Opuntia varieties from Texas. They observed that the predominant flavonoids in the PP with green peel were quercetin (43.2 μg/g fresh weight), isorhamnetin (24.1 μg/g fresh weight), and kaempferol (2.2 μg/g fresh weight) [13,66]. There is clear evidence that these compounds have an antioxidant power greater than several vitamins since phenolic compounds can delay the prooxidative effects in proteins, deoxyribonucleic acid (DNA), and lipids due to the generation of stable radicals [9,57].

In another study, whose objective was to investigate the amounts of total polyphenols and pigments from two PP cultivars of Moroccan origin, Khatabi et al. (2016) observed that the level of polyphenols was higher in the whole fruit than in its juice. They also concluded that the PP with red skin contained a higher quantity of polyphenols (15.34 mg/kg of juice and 17.81 mg/kg of whole fruit) than the yellow variety (15.03 mg/kg of juice and 15.03 mg/kg of whole fruit) [25].

Oniszczuk et al. (2020), executed research to determine the antioxidant properties and the content of polyphenolic compounds in PP. This study showed that PP was a rich source of phenolic compounds, particularly the benzoic acid derivatives (protocatechuic, syringic, 4-OH-benzoic, vanillic, gentisic, and salicylic), as well as the cinnamic acid derivatives (caffeic, trans-sinapic, cis-sinapic, β-coumaric, ferulic, isoferulic, m-coumaric, and 3,4-dimetoxycinnamic). In addition to this research, they produced a gluten-free pasta from rice-field bean flour that was enriched with various quantities of PP, and analysed this to determine its content of free phenolic acids, its antioxidant properties, and the sum of its polyphenols. The obtained results demonstrated that this gluten-free pasta supplemented with PP was a good source of natural antioxidants and could improve the quality of health and life of coeliac consumers [85].

Like flowers, fruits also contain betalains, and vacuolar pigments, which contain nitrogen and can be classified into betacyanins (such as betanin) and betaxanthines (such as indicaxanthin). The first gives a red colour and has absorbances at 540 nm; the latter provides a yellow colour and shows absorbances at 480 nm. In addition to these pigments justifying the range of colours available in the PP, they add important nutritional characteristics as they have higher antioxidant properties than those reported for ascorbic acid [5,6,13].

Sepúlveda et al. (2003) studied the betanin content of fourteen types of *Opuntia* from different regions of Chile. The results indicated significant variability of betanin concentrations in the fruits analysed (48.3 to 138.1 mg/100 g) [59]. In another study, Butera et al. (2002) investigated the existence of betalains and the antioxidant activity of aqueous extracts of red, yellow, and white PPs from cultivars from Sicily. Accordingly, with the results obtained, the yellow ecotype exhibited the highest amount of betalains, followed by the red and white ecotypes. Indicaxanthin represented about 99% of betalains in the white ecotype, while the betanin/indicaxanthin ratio varied from 1:8 (*m*/*m*) in the yellow ecotype to 2:1 (*m*/*m*) in the red ecotype [76]. Two other pigments in the PP are chlorophyll (green colouration) and carotenoids (orange colouration), although in smaller quantities.

Morales et al. (2009) carried out a study to characterise the pulps of two PP ecotypes (purple and orange), from Chile. Regarding the carotenoid content, they obtained 1.999 μg/g in the edible pulp of the purple ecotype and 0.984 μg/g in the edible pulp of the orange ecotype. In both ecotypes, β-carotene, lycopene, and lutein were detected [68].

All the pigments mentioned above make the fruits and their products attractive. However, their stability is the subject of continuous study. Betalains are soluble in water, and their stability is less affected by pH than anthocyanins, another class of natural red–purple pigments, being relatively stable in pH, between 3 and 7, which allows them to be used as additives in foods with low acidity and neutral pH, like dairy products [59,86]. On the other hand, betalains are more stable than chlorophylls under heat treatment and pH variation. In this way, the products of the purple ecotype tend to be more stable than those of the green ecotype [87].

Variations in the mineral content of PP can be attributed to different origins of this fruit, namely the total mineral content in the surface soils, the fraction that is bioavailable in the soil, the pH and texture of the soil, the presence of organic matter, the oxidation–reduction conditions, and the presence of clays. As a rule, the PP is an excellent source of minerals, such as potassium (217 mg/100 g), and is low in sodium (0.6 to 1.19 mg/100 g), which is beneficial for people with kidney problems and high blood pressure. It is also rich in calcium, phosphorus, and magnesium, with levels around 15.4 to 32.8 mg/100 g, 12.8 to 27.6 mg/100 g, and 11.5 to 16.1 mg/100 g, respectively [6,9,84].

In the case of calcium, it is necessary to carry out further studies on the bioavailability of this mineral due to the calcium oxalate crystals that can be found in the OFI that present a low absorption by the organism [59,84].

Aregahegn et al. (2013) determined the concentration of several minerals (Ca, Mg, Fe, Mn, Zn, Cu, Co, Cr, Ni, Cd, and Pb) by atomic absorption spectrometry in PPs collected in five different areas of Ethiopia. The average concentration value of each mineral was 283–564 μg/g of Ca, 187–386 μg/g of Mg, 3.03–4.50 μg/g of Fe, 1.33–4.03 μg/g of Mn, 2.45–7.32 μg/g of Zn, 0.85–1.95 μg/g of Cu, 0.174–0.295 μg/g of Co, 0.181–0.242 μg/g of Cr, and 0.330–0.856 µg/g of Ni. The toxic metals Cd and Pb were not detected in the samples [72].

El-Gharras et al. (2006) evaluated the effect of the state of maturation on the physicochemical properties of Moroccan PP, in three stages of maturation. Calcium increased from 111.11 mg/kg to 263 mg/kg [69]. These results were higher than those reported for the Mexican cultivars (110–170 mg/kg) [80] and were relatively lower than those reported for Chilean cultivars (154–328 mg/kg) [73]. The potassium content ranged from 963.9 to 2338 mg/kg [69], higher than that reported for Mexican cultivars (610–720 mg/kg) [80] and Chilean cultivars (12.8–27.6 mg/kg) [73]. The sodium concentration ranged from 50.78 to 236.78 mg/kg [69], with values comparable to those reported for the Mexican cultivars (120–160 mg/kg) [80]. There was no great variation in the copper content. The amount of zinc decreased from 0.62 to 0.29 mg/kg [69], being much lower than that reported for the Mexican cultivars (12–16 mg/kg) [80].

Due to the high water content in the PP pulp, its energy value varies between 31 and 50 kcal/100 g, comparable to other fruits such as apples, oranges, peaches, and pears [6].

### 3.3. Medicinal Properties

**Figure 3:**
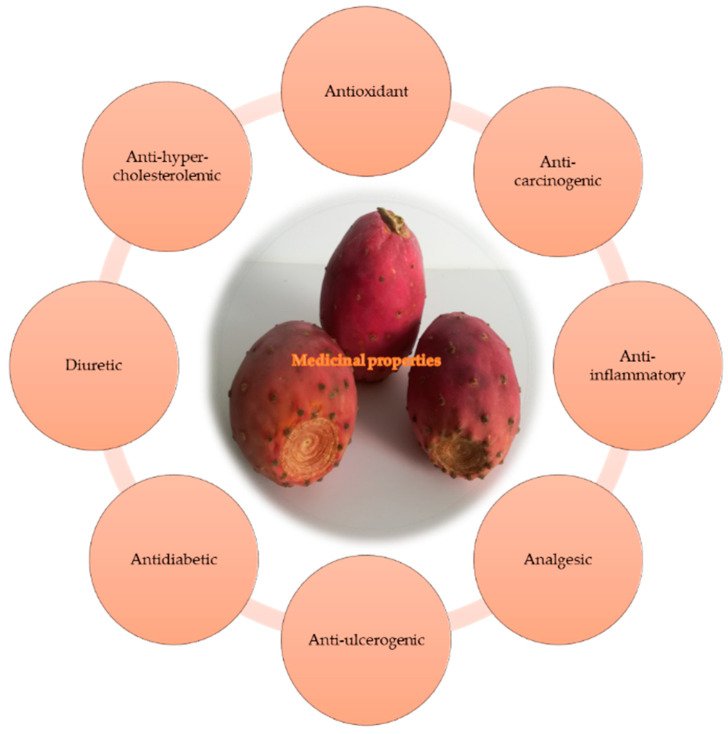
OFI’s medicinal properties [5,76,87,88,89,90,91,92].

According to numerous studies, a diet rich in fruits and vegetables is related to a lower incidence of cardiovascular diseases and some types of cancer [5], and this is no different for the OFI. Scientific research contains several studies that confirm that fruits and cladodes can be used as a source of nutrients and phytochemicals. In this way, OFI is valued not only for contributing to a healthy diet but also because it is rich in health-promoting substances used in the prophylaxis of various diseases [3,5,6]. Some of the medicinal properties of OFI discussed in this section are represented in Figure 3 [5,76,87,88,89,90,91,92].

In Latin America, cladodes are used in traditional medicine to treat bruises, burns, wounds, and infections. In Mexico, PP juice is valued as a laxative and diuretic, while the flowers are used for chest pains [28].

In general, the OFI has applications not only in preventing hangovers and in reducing lipid oxidation but also mainly in preventing the development of some types of cancer and reducing the risk of developing type 2 diabetes and cardiovascular disease, two of the most common causes of death [28].

As previously mentioned, the PP has a high antioxidant activity, attributed to ascorbic acid, carotenoids, flavonoids, polyphenols, and betalains [5,6,9,10,13,25,57,66,86]. The antioxidant activity of the PP is twice as high as that of other fruits, such as pears, apples, tomatoes, bananas, and white grapes, and has similar levels to red grapes and grapefruit [5]. This beneficial effect is generally attributed to the ability of these compounds to fight oxidative stress and modulate the activity of various enzymes and cell receivers [17].

Butera et al. (2002) analysed aqueous and methanolic red, yellow, and white PP extracts. This study revealed high antioxidant activity, in both the chemical and in vitro biological assays. The results concluded that betalains are one of this fruit’s most important antioxidant components. The white PP extract exhibited the greatest protection in all in vitro lipid oxidation models, and the acid ascorbic did not represent more than 40% of the antioxidant activity measured in the different extracts [76].

Dok-Go et al. (2003) evaluated flavonoids’ protective effects against oxidative neuronal damage induced in mouse cortical cells. Quercetin, dihydroquercetin, and quercetin 3-methyl ether were isolated from ethyl acetate fractions of fruits and cladodes and identified as neuroprotective compounds. Quercetin 3-methyl ether was the most potent of the isolated flavonoids [13,93].

Sirivardhana and Jeon (2004) demonstrated the antioxidant effects of the PP extract in inhibiting lipid peroxidation in oils and emulsions. The characterisation of these properties proved that the antioxidant compounds present in PP are stable, offering a natural source for stabilising edible oils [94].

Fernández-Lopéz et al. (2010) investigated the presence of antioxidant compounds and the respective in vitro antioxidant capacity in extracts of three Spanish species of *Opuntia* with red peel. This study confirmed the potential of the three extracts as important sources of bioactive compounds, including ascorbic acid, carotenoids, taurine, and flavonoids. The OFI extract contained the highest antioxidant capacity and taurine content [3,65].

Lee et al. (2002) evaluated an ethanol extract from cladodes to determine the mechanisms of its antioxidant activity. The extract showed elimination activity dose-dependent free radicals, including 2,2-diphenyl-1-picrylhydrazyl radicals (DPPH^•^), the superoxide anion (O_2_^•−^), and hydroxyl radicals (OH^•^), using different systems of rehearsal. This extract was also considered effective in protecting plasmids against chain rupture, induced by hydroxyl radicals in the Fenton reaction. The researchers identified a high quantity of phenolic compounds responsible for the extract’s antioxidant properties [95].

El-Hawary et al. (2019) characterised the polyphenolic constituents of extracts from OFI cladodes, fruit peel and fruit pulp, and investigated their antioxidant and neuroprotective activities. They characterised 37 secondary metabolites using HPLC-MS/MS, and in DPPH assays the extracts exhibited significant antioxidant activities. In vivo, the extracts demonstrated considerable neuroprotective activity against AlCl_3_-induced neurotoxicity (Alzheimer’s condition): OFI extracts significantly decreased the elevated brain levels of proinflammatory cytokines and increased anti-inflammatory cytokine and monoamine neurotransmitters compared with the positive control group. Thus, they concluded that OFI responds to oxidative stress and may be a good candidate for the treatment of several neurological disorders [96].

Diabetes mellitus is another of the biggest problems in public health, characterised by defects in insulin secretion, insulin action, or both [87]. Natural products have long been used in traditional medicine for diabetes. In Mexico, local healers recommend drinking fresh juice from cladodes, and fresh, fried, or grilled cladodes to treat type 2 diabetes [97].

Several studies demonstrate the hypoglycaemic and antidiabetic activity of extracts of OFI. Butterweck et al. (2001) studied the effects on blood glucose concentration and plasma insulin in normal mice of an aqueous extract prepared from cladodes and a mixture of cladode peel and fruit. The results showed that the aqueous extracts reduced blood glucose levels and increased plasma insulin when given orally at low doses (6 mg/kg). Additionally, the peel mixture directly affected pancreatic β-cells [87].

Alarcon-Aguilar et al. (2003) investigated whether polysaccharides isolated from OFI had hypoglycaemic effects. Traditional OFI preparations were evaluated in temporarily hyperglycaemic rabbits, alloxan-diabetic rabbits, regular volunteers, and type 2 diabetic patients. The results suggest that the hypoglycaemic effect produced by the OFI can be explained by a mechanism that reduces the intestinal absorption of glucose due to some effect of the SDF present in the OFI. Clinical trials indicate that the OFI helps stabilize blood sugar levels, effectively treating type 2 diabetes [97]. One of the possible mechanisms of action for the hypoglycaemic activity of OFI is due to the high content of SDF present in this plant. These increase the viscosity in the intestinal tract, slowing down or reducing the absorption of sugar, and may also reduce the fat absorption in the intestine and contribute to improving lipid profiles and weight loss. The SDF content is not the only mechanism of action since fasting glucose levels in the blood are also affected [98].

A study by Ennouri et al. (2005), whose objective was to determine the fatty acids from PP seed oil and evaluate the effects of a supplementary diet with this oil (25 mg/kg) in mice, proved that this food supplement reduced the concentration of serum glucose, and was associated with the formation of glycogen in the hepatic and skeletal muscles. These observations were explained by a potential induction of insulin secretion, which stimulates the conversion of glucose to glycogen [13,99].

Current treatment strategies for type 2 diabetes generally involve pharmacological treatment aimed at stimulating insulin secretion or increasing sensitivity to insulin. Thus, the results obtained in these studies are promising since there is an opportunity to develop pharmaceutical treatments to improve B cell function and reduce insulin resistance, which is crucial to improving metabolic control and delaying the development of diabetic complications [87].

Hwang et al. (2017) investigated the antidiabetic effects of aqueous extracts of cladodes in preventing and treating diabetes. In this way, they performed glucose tolerance assays, and measured α-glucosidase activity and dietary fibre content in streptozotocin-induced diabetic mice. Based on the results obtained, the authors suggested that the investigated extracts could be considered as a food supplement in the prevention and/or treatment of diabetes [100].

Chemoprevention is an approach in which chemical agents prevent, reverse, or block the onset of cancer in certain risk groups. Although it is a promising technique in some epithelial cancers, available preventive agents currently are limited, expensive, and have side effects. Therefore, some natural products, such as grape seeds, green tea, and some herbs, which demonstrate anticancer effects, have been investigated intensively for their possible effects [9,29,48,88,101].

Zou et al. (2005) investigated the aqueous extracts of PP from Arizona in cancer cells from the ovary, cervix, and bladder. Depending on the dose and time of treatment, these extracts can inhibit the in vitro proliferation of tumours, comparable to the inhibition demonstrated by the synthetic retinoid N-(4-hydroxyphenyl) retinamide (4-HPR), which is a chemopreventive agent widely used in the chemoprevention of ovarian cancer. The inhibition of the in vitro growth of cancer cells was associated with an increase in apoptotic cells and cell cycle arrest in the G1 phase. The mechanism of action of the anticancer effect of PP extracts is not yet understood, but it appears to be dependent on the P53 pathway, which is the primary tumour suppressor. This study also demonstrated that the extracts of PP could also suppress ovarian cancer growth in the in vivo mouse model. The intraperitoneal administration of the PP extract solution did not affect the mice’s body weight, indicating that the extract showed no toxic effects [88].

Several assays suggest that OFI contains anti-hyperlipidemic and anti-hypercholesterolaemic properties, as it reduces blood cholesterol levels and modifies the composition of low-density lipoproteins (LDLs), which are considered an atherogenic risk due to their affinity for cholesterol in corporation and resultant deposits (atherosclerotic plaques) on blood vessel walls [102].

In a series of studies with guinea pigs, Fernandez et al. (1990, 1992, and 1994) showed that the reduction in blood lipid concentrations, triggered by pectin isolated from OFI, was due to binding to bile acids. In this way, the reduction of bile absorption in the colon alters the enterohepatic circulation [103,104,105].

A study by Wolfram et al. (2003) showed that daily consumption of 250 g of PP pulp reduced the risk of thrombosis in patients suffering from hyperlipidaemia and diabetes [20]. Galati et al. (2003) studied the influence of daily administration of cladodes lyophilised in the lipid metabolism of hypercholesterolaemic mice, having evaluated the levels of cholesterol, high-density lipoprotein (HDL), LDL and triglycerides. The treatment was more effective after 30 days, with statistically significant reductions in cholesterol, LDL, and triglyceride levels in plasma. The effects are probably due to the high fibre content from cladodes, but other active ingredients may work together with this one [92].

Ennouri et al. (2005) observed a decrease in cholesterol and LDL concentrations in blood, with no changes in HDL concentrations, after adding PP seeds to the diet of mice. These results support the nutritional value of OFI as a natural source of edible oil that contains essential fatty acids [13,99].

LDL particle size has appeared as a significant predictor of cardiovascular disease and progression of coronary heart disease. Therefore, the quantity and quality of particles, mainly small and dense LDL (sdLDL), are essential in determining cardiovascular disease risk. Giglio et al. (2020) did the first intervention study suggesting that pasta enriched with an OFI extract might have beneficial effects on some metabolic parameters and the LDL particle size and distribution, reducing atherogenic sdLDL (−45%). For one month, 49 patients with one or two criteria for the metabolic syndrome consumed 500 g of pasta supplemented with 3% OFI extract weekly. After one month of pasta supplementation, plasma glucose, triglycerides, plasma creatinine, urea, and aspartate transaminase significantly decreased [106].

The severity of hangovers from alcohol consumption may be related to the inflammation induced by impurities in alcoholic beverages and by-products of alcohol metabolism. The best hangover prevention is, of course, abstinence from alcohol. Still, it has never been shown to stop alcohol consumption effectively, and no evidence indicates that symptom relief results in increased consumption. In addition to the various symptoms (nausea, headache, dry mouth, weakness, tremors, diarrhoea, and dizziness) associated with a hangover, it also poses a risk of injury in the workplace, which can lead to cognitive changes and reduce dexterity, and visual and spatial awareness. To evaluate the effect of extracts from the PP peel in the reduction of the symptoms of a hangover from average alcohol consumption, Wiese et al. (2004) conducted a randomised crossover study with 64 healthy young adults. The results obtained showed that the analysed extract had a moderate effect in reducing hangover symptoms, inhibiting the production of inflammatory mediators, accelerating the synthesis of shock protein heat during periods of stress, and decreasing oxidative damage [90].

Park et al. (1998) prepared ethanol extracts from the fruit and cladodes to evaluate their pharmacological effects in mice. Both extracts showed an analgesic effect like acetylsalicylic acid, even at the highest doses, without toxic effects. Furthermore, the extracts suppressed the release of β-glucuronidase, one of the lysosomal enzymes released by inflammatory cells infiltrating tissue damaged by phagocytosis. This result indicates that the extracts’ effects may inhibit the release of inflammatory mediators. A protective effect was also observed in the layers of the gastric mucosa, suggesting that the extracts contained a protective effect against gastric lesions [2,3,21,105].

Park et al. (2001) evaluated several methanolic extracts of OFI cladodes for their ability to treat wounds in mice. The n-hexane and ethyl acetate fractions showed significant activity when administered topically. These results demonstrated that methanolic extracts showed an anti-inflammatory action and advantages in using OFI in wound healing [3,107,108]. Park et al. (2001) continued to study the methanolic extracts of cladodes through a model of induced chronic inflammation in mice, and the anti-inflammatory principle active was isolated and identified as β-sitosterol. However, its activity appears relatively lower than hydrocortisone’s [3,109].

The effects of PP powder were investigated by Lee et al. (2001), regarding gastric injuries and ulcers in mice. The results indicated an inhibition of the gastric lesions induced by hydrochloric acid/ethanol and hydrochloric acid/acetylsalicylic acid. The rate of gastric juice secretion and the pH value remained constant. These data showed that OFI has an inhibitory action on gastric lesions in mice [91].

Galati et al. (2001, 2002, and 2003) also confirmed these results. They studied the effect of administering lyophilised cladodes, which showed significant anti-ulcerogenic activity in the experimental model, on ethanol-induced experimental ulcers in mice. The curative and preventive treatments showed different results. In the curative treatment, gastric mucosal epithelial cells appeared injured. The acute treatment with lyophilised cladodes probably did not have time to restore mucosal defence factors upon ethanol induction [110]. Nonetheless, when lyophilised cladodes were administered as a preventive therapy, these maintained the gastric mucosa under normal conditions, preventing the mucus dissolution caused by ethanol and favouring mucus production [89].

In another study, Galati et al. (2003) inferred that the preventive administration of PP juice inhibited the ulcerogenic activity of ethanol in mice. The obtained results indicated increased mucus production and restoration of the standard mucosal structure. These studies demonstrated an evident protective activity of cladodes and the juice of the PP against ethanol-induced ulcers [26].

Similar results were obtained by Khémiri et al. (2019). They carried out a study to investigate the preventive and curative effects of the PP seed oil in an ethanol-induced gastric ulcer model in mice. The oil showed high efficiency in protecting the structure and function of the gastric mucosa against damage caused by ethanol ingestion. Mucus production was stimulated, the volume of gastric juice was reduced, and its pH increased. They also concluded that the fatty acids in the oil, mainly unsaturated and triglycerides, contributed to the repair of the lipid bilayer of the cell membrane during the gastric ulcer healing process [111]. The protective activity of PP juice, namely betanin, was evaluated against acute gastric disorders caused by induced stress in mice.

Kim et al. (2012) observed that the pre-treatment made with lyophilised powder containing PP juice and maltodextrin significantly reduced lesions. Furthermore, it effectively prevented the decrease in gastric mucus content, which may be related to the production of pro-inflammatory cytokines [112].

Galati et al. (2002) investigated the acute and chronic diuretic effects of flowers, fruits, and cladode extracts in mice. The flower and cladode extracts increased diuresis but did not influence the uric acid cycle. On the other hand, the fruit extract showed diuretic and anti-uric activity. All extracts analysed showed a modest but insignificant increase in sodium and potassium urinary levels. There are some possibilities for the observed diuretic effect, which still needs to be investigated. On the one hand, these natural compounds can act synergistically or individually, promoting initial vasodilation. On the other hand, it is possible that the extracts manifest an accumulation of several substances and/or it is due to secondary active metabolites. Another possibility for this effect may be due to indirect changes in some physiological parameters before blood filtration. The anti-uric effect of the PP infusion cannot be explained only by increased diuresis or urinary excretion, which may be linked to an alteration of some enzymatic activity [3,92].

Monoamine oxidases (MAOs) are enzymes whose function is to degrade monoamines, preventing them from accumulating (in the case of endogenous monoamines) or generating undesirable effects (in the case of exogenous monoamines), being involved in the catabolism of catecholamines. These constitute a class of chemical neurotransmitters and hormones that occupy critical positions in regulating physiological processes and developing neurological, psychiatric, endocrine, and cardiovascular diseases [113].

Han et al. (2001) performed several assays, including anticoagulant activities, dopamine β-hydroxylase, and MAO, in several extracts of cladodes and fruits of Korean OFI. Methyl esters derived from organic acids have been identified as MAO inhibitors. The aqueous extracts showed the lowest inhibitory activity, followed by the n-butanol fraction and the hexane extract, while the ethyl acetate fraction exerted the most significant inhibitory action [2,114].

### 3.4. Applications and Agro-Industrial Uses

#### Applications

**Figure 4:**
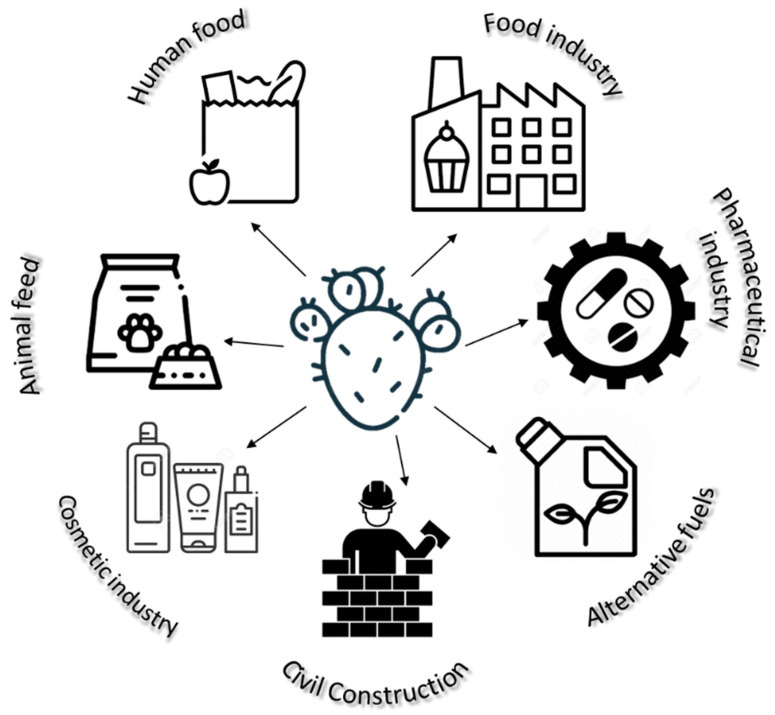
Applications of OFI fruits and cladodes [2,5,17,18].

Considering the nutritional and pharmacological properties of OFI, it has several applications, both in human and animal nutrition, in the food, pharmaceutical and cosmetic industries. OFI is also used in civil construction and alternative fuels (Figure 4) [2,5,17,18].

The most notorious application of OFI is consuming the fruit after peeling. In some countries, such as Mexico and Chile, PP juice is consumed at home, in restaurants, or in local stores [115]. In these countries, the PP fermented juice, without the seeds, is known as a beer called “colonche” [116].

OFI flowers are used for teas, and cladodes are consumed as fresh vegetables, being used as an ingredient in several dishes, including sauces, salads, soups, snacks, drinks, and desserts [5,28].

The properties of OFI make it advantageous for a wide variety of products in the food industry. Beyond the fresh fruit, minimally processed fruit, dehydrated or preserved fruit, we can find in the market juices, fermented drinks, liquid sweeteners, pulps (frozen or dry), gums or gels, jams or jellies, flours, seeds, natural dyes, dietary fibres, and thickeners [6,8,10,16,117,118,119].

In addition to human food, the OFI also contributes to animal feed. Cladodes and fruits are profitable feed for ruminant animals, especially when pasture supply is lower and of low quality [5,116].

OFI also has advantages in the pharmaceutical industry due to its medicinal properties. Powdered extracts of PP, cladodes, and flowers are commercialised in capsules to protect the gastric mucosa, regulate weight, regulate blood sugar, or increase fibre intake. Cladode-based gels have a cooling effect, like aloe vera preparations, relieving the skin and contributing to wound healing [2,6].

Another OFI application is in the cosmetic industry, as the PP, cladodes juices and the seed oil can be found in shampoos, conditioners, creams, lotions, soaps, face masks, and sunscreens [2,6,117].

Ammar et al. (2012 and 2015) studied the presence of bioactive substances with antimicrobial action on the OFI flowers. This investigation pointed to the feasibility of using flower extracts against various microorganisms, such as *Pseudomonas aeruginosa*, *Staphylococcus aureus*, and *Escherichia coli*, and these have been recommended as a preservative in multiple fields of application, including agri-food, cosmetics, and pharmaceutical [3,14,120].

Typically, OFI is used as a hedge or as an ornamental plant. Still, it also has several environmental advantages, namely in protecting fauna, combating desertification, and as a source of nectar for bees [7,121]. OFI is one of the most efficient plants in water use, protecting against soil erosion [121].

In a study carried out by Barka et al. (2013), OFI in an aqueous solution showed a bioabsorption capacity for cadmium and lead, and can be considered as an effective, low-cost, natural, and ecological absorbent for these two metals (a process known as phytoremediation), being an alternative to the current expensive methods of removal of metals from wastewater [122]. In addition to the adsorption capacity, OFI also presents compounds with antimicrobial activity and coagulant properties, which can be used to decontaminate river water and wastewater [4,18].

The coagulant, thickener/gelling, and polyelectrolytic activities of OFI are mainly to its polygalacturonic acid content. The clotting activity can be due to carbohydrates and several phytochemicals, namely phenols, carotenoids, flavonoids, betalains, vitamins, minerals, amino acids, amines, organic acids, lipids, and terpenes. These compounds have diverse chemical structures and functional groups, allowing the adsorption of various contaminants [18].

OFI plantations are also crucial for reproducing cochineals (*Dactylopius coccus*), an insect genus that thrives on this plant. These insects are important in biological control against invasive cacti and are a source of carminic acid, a red pigment used in the colouring of foods, cosmetics, pharmaceuticals, and fabrics. This pigment has been investigated for its antioxidants and antimicrobials, which point to potential applications in immunology and wastewater treatment [3,5,64].

OFI also has applications in the civil construction area, as a protective agent against corrosion, due to mucilage, and as a building material to improve stability and compressibility [2,123].

Through the fermentation of cladodes, OFI has advantages in producing alternative fuels, namely biogas, a viable and essential form of energy in agricultural and rural areas. Biogas is obtained from the transformation of organic waste through anaerobic digestion. This plant is recommended as an alternative energy source since it has a high potential to produce biomass [2,5,124].

## 4. Physical, Chemical, and Microbiological Changes in Prickly Pear

PP has a short shelf life of about 3 to 4 weeks, a pH of 5.6 to 6.5, and a low acid content (0.05 to 0.18% citric acid equivalent), which compromises its prolonged storage and distribution locally and worldwide [9]. Considering its physicochemical composition, the PP is susceptible to physical, chemical, and biological modifications, and there may be losses of some food constituents, especially nutrients [7]. In Mexico, it is estimated that during the commercialisation of PP there are losses of about 15% of the harvested fruits [11,125].

In general, harvested fruits’ deterioration rate is proportional to their breathing rate. The PP is a non-climacteric fruit with a low respiration rate at 20 °C (20 mL CO_2_/kg/h) and a reduced ethylene production (0.2 μL C_2_H_4_/kg/h). These values are similar to oranges [11,125].

During the conservation of the PP, the physical damages are the leading cause of the alteration of this fruit. These changes are due to its physiology and post-harvest handling, mainly due to removing glochidia [7]. PP susceptibility to physical injury increases with maturity [5]. The discolouration is one of the main post-harvest problems and is caused by the oxidation of phenolic compounds catalysed by the enzymatic actions of polyphenol oxidase (PPO) [126]. There are differences in susceptibility between the various ecotypes because the green PP appears to be more sensitive to this problem [11,125]. In addition to the visual aspect, the discolouration of the fruits leads to changes in flavour and losses in the nutritional quality of the fresh fruits, which affects consumer acceptability [11,125,126].

PPs are very sensitive to water loss, making them perishable [127]. The PP begin to show signs of rotting nine days after harvest, and twenty days after harvest PP shows water losses of between 70 and 80% [8]. These losses can also be justified by the damage caused by the fruit fly. The larvae in the damaged fruits can develop and evolve to the adult state, infecting the fruits that are not damaged and stored [7].

OFI is a tropical plant that can suffer cold injuries and damage caused by temperatures above freezing (0 °C). Flowers and fruits are more susceptible to this damage. Leaves may turn purple or reddish and, in some cases, wither. The sensitivity OFI presents to this type of lesion varies according to the cultivar, the fruit maturity, the environmental conditions, and the storage humidity. Usually, these lesions are not evident during cold storage, appearing during commercialisation when the fruits are transferred to higher temperatures [11,125]. Therefore, the fruits can be refrigerated for a maximum period of two months at temperatures of 0 ± 0.5 °C and 85 to 90% relative humidity [6].

Like most non-climacteric fruits, the PP does not contain starch as a reserve of carbohydrates. Therefore, after harvesting, the physicochemical parameters that tend to decrease are total soluble solids (TSS), sugars, and organic acids. If the PPs are stored at room temperature, the ascorbic acid content will rapidly decrease, but this content becomes relatively stable at low temperatures, despite the high pH [5,11,125].

The pH, acid content, and TSS values presented by PPs make their pulp a very attractive medium for the growth of microorganisms, which limits their useful shelf life in the fresh state [84,127,128]. Microbiological damage is favoured mainly by pathogenic microorganisms, namely *Fusarium* spp., *Alternaria* spp., *Chlamydomyces* spp., and *Penicillium* spp. [8]. In the case of purple PPs, the microbiological damage is minimised because betalains have greater stability than chlorophylls under the same heat treatments and pH variations [6].

Various means to minimise nutritional and sensory losses and microbiological growth include the storage of the PP in refrigerated conditions, guaranteeing adequate sanitary conditions, packaging in an active or passive modified atmosphere, and using edible coatings [6,7]. In addition to conservation processes, transformation processes are also essential to avoid post-harvest losses and increase the lifetime of the PP, although some technologies modify some fruit constituents [7,17].

## 5. Preservation Methods of Prickly Pear

The agro-industrial applications that can be applied to fruits and vegetables aim to make full use of them, reduce production losses and enable their valorisation through appropriate processing to diversify the offer of new products, with an extended useful life and with a reduction of transport, packaging, and storage costs [7].

After harvest, refrigeration is the most used technique to extend PP shelf life, and delay the physicochemical and microbiological changes mentioned previously. These procedures’ main objectives are to reduce transpiration and respiration rates, increase fruit tolerance to cooling temperatures, and prevent microbial development [5].

**Figure 5:**
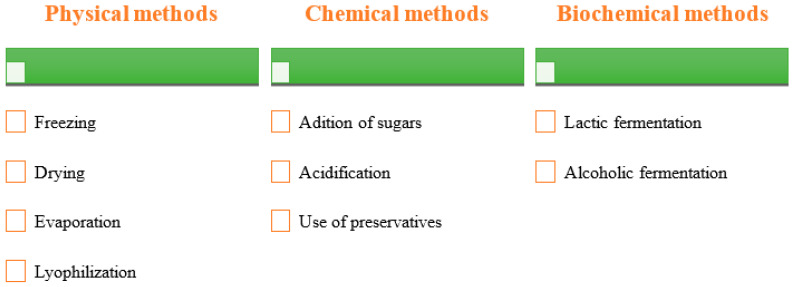
Conservation and transformation methods applied to the PP [6].

Conservation and transformation systems include technologies based on physical, chemical, or biochemical methods (Figure 5). Physical methods include those that use heat transfer as a means of preservation, immobilisation of water, or the reduction of water activity (a_w_). Examples of physical methods applied to food preservation are freezing, drying, evaporation, and lyophilisation. Chemical processes include the addition of sugars, acidification, and the use of preservatives. Biochemical methods are based on lactic or alcoholic fermentations [6].

**Figure 6:**
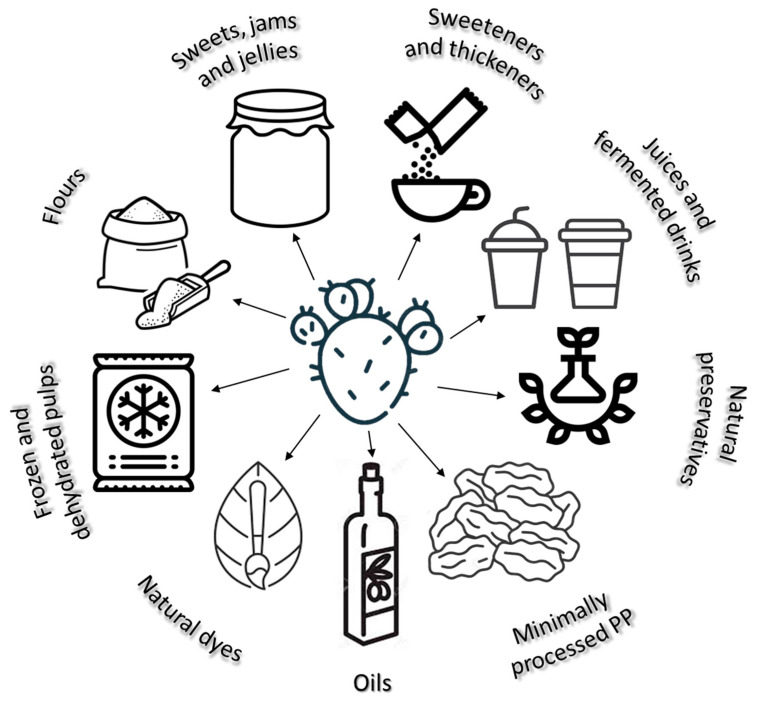
Products from conservation and transformation methods applied to the PP [5,6,7,8,129,130].

Some of the products from the various processing methods mentioned above are fresh preserved PPs, minimally processed PPs, dehydrated PPs (dry or osmotically dehydrated), preserves, juices, fermented beverages, sweetener liquid, the pulp (frozen or dehydrated), gums or gels, jams or jellies, flours, seed oil, natural dyes, dietary fibres, and thickeners (Figure 6) [5,6,7,8,129,130]. Considering the effects of seasonality and the PP distribution, selecting the most suitable processing method depends on the intended purpose and the conservation to be applied [7].

As the handling of the raw material affects the quality of the final product, some steps must be carried out before the conservation phase, which are common to all the food industries: reception of raw materials, cleaning, selection, and washing of the sample. Usually, the cleaning step is mechanical, with the fruits going through rotating brushes to eliminate the glochidia smoothly and without damaging the epidermis of the fruits. Then, the fruits are selected on a purpose-specific basis. The fruits that are damaged, rotten, or inadequately ripe are removed. Subsequently, the fruits are washed with potable water and, if possible, chlorinated at room temperature to reduce the microbial load. If a transformation method is applied, in addition to these steps, the peeling and cutting operations are also necessary [7,131,132,133].

The harvest and post-harvest handling of the PP must be careful to avoid physical damage and contamination. The fruits must be whole and in excellent maturation, a homogeneous size, in good physical condition, and with an absence of defects [7]. The removal of glochidia is the most critical post-harvest operation, being performed mechanically. The fruit selection is made manually by operators, who must wear gloves and visual protection. Mechanical cleaning is carried out by passing the fruit through a series of brush swivels with firm hairs, which smoothly eliminate the glochidia without damaging the fruit epidermis [7,133]. Subsequently, the fruits are selected, washed, and sanitised. If it is necessary to carry out the peeling of the PP, this is accomplished manually with the use of knives. First, the ends of the fruit are cut, followed by a longitudinal cut, so that the epidermis is extracted in one piece [5,7].

Fresh preserved PP is obtained by waxing, by immersion or spraying of wax, to control the loss of water by transpiration, reduce the intensity of the fruit’s gas exchange, improve its appearance, and prolong its conservation [8]. This product’s most used packaging is wood, plastic, or card. After packaging, the PP must be stored between 5 and 8 °C. The effectiveness of this type of conservation depends on several factors, such as storage time, type of packaging, and time of harvest [7].

According to the *Codex Alimentarius*, the fresh PP commercialisation must comply with several minimum quality requirements, such as being whole, healthy, clean, devoid of glochidia, free from pest damage, free from abnormal external humidity, free from damage caused by low temperatures, free from any extraneous aroma and taste, and free from pronounced stains, and have a fresh appearance, a firm consistency, and a satisfactory degree of ripeness. PPs can be classified into three categories [47]:

− “Extra” category, which includes superior quality fruits that do not contain defects, except superficial ones, that are very light, and that do not affect the general appearance of the product and its state of conservation;
− Category I, which includes good quality fruits, with defects allowed being light in shape, colour, and skin, such as spots, scabs, and other blemishes on the surface areas, provided that the total area affected does not exceed 4% and that the defects do not affect the pulp of the fruit;
− Category II includes fruits that do not fit the categories above, with the surface affected by the defects, referred to in category I, not exceeding 8%.

As an alternative and without compromising the nutritional qualities of the fruit, there are minimally processed PPs, with characteristics like those of fresh PPs, but with a longer lifetime. After washing the fruits, they can be peeled and cut, and then subjected to natural or artificial drying [7]. Despite the advantages of this processing, the physical damage caused to plant tissues makes these products more perishable than when intact due to the acceleration of metabolism from cutting [129]. To avoid this problem, these products are packaged in modified atmosphere packaging (MAP) and stored at refrigerated temperatures (5 to 6 °C) [7].

**Figure 7:**
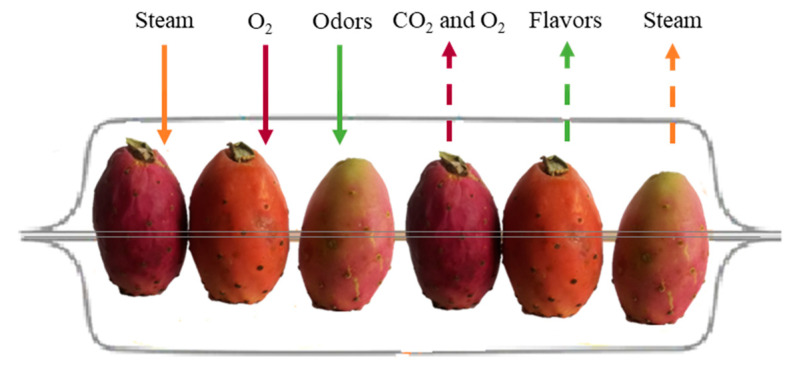
Transfers that occur through modified atmosphere packaging [7].

In MAP, the modification of the atmosphere is the result of breathing in the packaged product depending on the permeability of the packaging and temperature (passive modification) or due to the injection of a gaseous mixture in the free space of the package, so that the atmosphere is determined by the interaction between the product, the packaging, and the environment (active modification) [12,132]. MAP implies a decrease in the concentration of O_2_ and/or an increase in CO_2_ levels, reducing the rate of respiration, production, and action of ethylene, the degradation of chlorophyll, the loss of texture, and the delay in the maturation of the product [134]. Decreased O_2_ and high CO_2_ concentrations can also reduce or inhibit the food spoilage and the growth of pathogenic microorganisms [135]. The main constituents of the films used in MAP are low-density polyethylene (LDPE), polypropylene (PP), polyvinyl chloride (PVC), polyethylene terephthalate (PET), polystyrene (PS), and cellulose. The increase in the product’s useful lifetime is due to the balance between it and the packaging. Therefore, it is important to adjust the permeability of the film to the O_2_ and CO_2_ concentrations, according to the respiratory rate of the PP, represented in Figure 7 [7].

Piga et al. (2003) investigated possible changes in ascorbic acid and polyphenols levels in whole PP, manually peeled and packaged in passive MAP. The mass decrease of the PP was low, with a maximum of 0.15 g/100 g, after nine days at 4 °C. The minimal processing applied to the PP did not indicate a significant decrease in ascorbic acid and polyphenols levels nor in their antioxidant capacity. Despite being significantly altered during storage, other parameters, such as acidity and pH, did not harm sensory parameters [131].

Inglese et al. (2002) mentioned using polyethylene films in PP stored at 6 °C for six weeks and commercialised at 20 °C [136]. Corbo et al. (2004) found that PP storage at 5% O_2_ and 30% CO_2_ caused a selective suppression of the growth of different microbial populations [137].

Cefola et al. (2011) studied the effects of different storage temperatures and modified atmospheric conditions on commercialising ready-to-eat PP. The storage at 4 °C, both in passive and active atmospheres (10 kPa O_2_ and 10 kPa CO_2_), improved marketability by 30%, while storage at 8 °C caused a reduction in the partial pressure of O_2_ in the MAP due to the increase in the metabolic activity of fruits, which contributed to the loss of commercialisation of the PP. The researchers concluded that it is possible to store PP in the yellow-green maturation phase, in active or passive MAP, at 4 °C for 9 days [128].

A complement to MAP is the application of edible coatings, which are thin pieces of edible material formed on a food surface or placed between food components [138]. Edible coatings applied to whole fruit and/or cut fruit can control water loss, delay ripening and colour changes, improve appearance and texture, prolong the lifetime, and create a barrier against various hazards. In addition to these advantages, these coatings can be used as carriers of antimicrobials or antioxidants. Examples of edible coatings are proteins and polysaccharides from renewable sources, such as starch, chitosan, carrageenan, alginates, soy proteins, corn zein, wheat gluten, casein, egg albumin, and fish proteins [127].

Del Nobile et al. (2009) tested different packaging designed to prolong the lifetime of minimally processed PP. Fresh fruits were coated with sodium alginate, agar-based gel, and fish-protein-based gel. Then they were wrapped in a film and biodegradable material. The results indicated that only sodium alginate coating extended the shelf life of minimally processed PP to about 13 days, responding to an increase of about 40% compared with the control sample. The pH values of all samples steadily decreased during storage, probably due to microbial fermentation, mainly attributable to yeasts. These results are also positive from an environmental point of view, reinforcing the need to replace synthetic materials with biodegradable films in various packaged foods [127].

Ochoa-Velasco et al. (2014) conducted a study to assess the effect of chitosan coatings containing different concentrations of acetic acid on the physicochemical, antioxidant, microbiological, and sensory characteristics of white- and red-peeled PP. Chitosan is produced through the deacetylation of chitin. It is a natural cellulose-like compound widely used for covering fruits and vegetables due to its ability to inhibit the growth of pathogenic microorganisms. The chitosan coatings form a semipermeable barrier capable of controlling gases and moisture. PPs treated with chitosan, containing 1% of acetic acid and stored at 4 °C and 85% relative humidity, maintained their quality for 16 days, while chitosan-coated PPs, including 2.5% of acetic acid, did not appreciate well due to the acidity present. Chitosan coatings retarded microbial growth in both PP varieties, regardless of the amount of acetic acid used. The main factor that limited the validity period of the white PP was the moisture loss; for the red PP, it was the loss of firmness [138].

Due to the difficulty in peeling PP, there is a great demand in the market for fresh-cut PP, i.e., ready-to-eat PP. As little information has been published about the conditions necessary for the post-harvest storage of ready-to-eat PP, Kahramanoğlu et al. (2020) selected five biomaterials (*aloe vera* gel, *Portucala oleraceae* extract, *Vitis vinifera* leaf, cactus gel, and jam) to coat the fresh-cut PP. The results suggested that the duration of post-harvest storage of ready-to-eat PP could be extended using the different biomaterials evaluated. In this context, they found that the *aloe vera* gel and the cactus gel were more effective in protecting against mass loss and microbial spoilage in the quality sensory input and changes in TSS concentration [126].

Dehydration is another food preservation method that has been used for centuries. This alternative consists of eliminating water by evaporation to reduce the risks of microbiological contamination and avoid chemical reactions, allowing the preservation of the fruit’s characteristics and to increase its lifetime at room temperature [7]. Dehydration is based on the reduction of a_w_, which is a measure that represents the amount of free water or available water in a food. Water availability in plant tissues is variable and depends mainly on the composition of the fruits, since some components, such as hydrocolloids, have a more remarkable ability to retain water [5]. Fresh fruits and vegetables have an a_w_ close to 1.0, hence their susceptibility to microbial attack and rapid perishability [6]. Microbial growth can be controlled by decreasing a_w_, because each microorganism has a critical a_w_, below which it cannot multiply [5]. Beyond the indicated advantages, dehydrated products generally do not contain additives, and are considered safe natural foods [6].

After the post-harvest stages, inherent to any technological process and mentioned above, the PP can be cut and dried at 60 °C to reduce the final fruit moisture content (<12%). After packaging, the dehydrated PP must be kept in a cool, dry place and protected from light. The conservation period is at least six months and can reach up to a year, depending on the moisture content of the air, after drying [7].

As an alternative to the traditional dehydration process (drying), there is osmotic dehydration (OD). This process consists of the partial removal of water by pressure caused when the product, whole or in pieces, is placed in contact with a hypertonic solution of sugars, without phase change, due to the difference in osmotic potential that checks between the products and the dehydrating hypertonic solution. In addition to its low-cost energy, it is a suitable method for all production scales, being an alternative technology to reduce post-harvest losses [43]. The final product must be packed in containers impermeable to gases and water vapour and kept in the dark at room temperature. In general, drying does not change the flavour of the PP. The OD increases the strength of the fruit structure and improves the final product’s flavour and colour. Despite these advantages, these alternatives have the same disadvantage, which is the hardening of excess pulp. As the PP has many seeds, which is evident with water removal, the final product becomes difficult to chew [7].

We can obtain dry mesocarp or dehydrated pulp to circumvent the inconvenience of seeds. In the case of dry mesocarp, after cutting the ends of the already peeled PP, the mesocarp is removed by longitudinally cutting it [5]. This is cut into strips and subjected to the drying process at 60 °C. The final product does not contain seeds and has a pleasant taste and a good texture. Its conservation is at least six months and it can be implemented in cereal flakes or in various snacks [7]. In the case of PP pulp, this is obtained through the pulping of the fruit, carried out through a sieve, intending to separate the pulp from the rind, fibres, and seeds [5]. The seedless pulp undergoes homogenisation, after which it is subjected to a formulation, with adjustment of the TSS content. After the pulp is processed, it has a porous appearance, with tiny rudimentary seeds, sweet flavour and light acidity, and is ready for consumption or to produce diversified, value-added products through other technological processes [7]. One of these products is dehydrated pulp that can be processed or mixed with other fruits, such as apples or quinces, to give body to the product without influencing the final taste [5]. The mixture is subjected to a drying temperature of about 60 °C, with ventilation, until a dehydrated chewable product is obtained. After dehydration of the pulp, with about 10 to 15% of humidity, it is cut into different sizes and thickness strips, serving as a raw material to produce various snacks, muesli, and other food products [7]. This approach has had a substantial degree of acceptance at the level of gourmet products, being considered as a ready-to-eat food consumed and available throughout the year [5]. The most suitable packaging for these products is plastic, impermeable to light and water vapour, and then placed in cardboard boxes and kept at room temperature [7].

El-Samahy et al. (2007) carried out a study with the dehydrated pulp of yellow–orange PP, to evaluate different drying temperatures (60 °C and 70 °C) and sucrose concentrations (0, 1, 2, 3, 4, 5, and 10%). The pulp was spread to a thickness of 10 mm and dehydrated in an air convection oven for 44 h. The final product consisted of dry PP leaves, which were organoleptically evaluated. The products with the best acceptance level were those prepared with 2 and 3% sucrose [139].

Within the dehydration techniques, a method widely used for drying pulps and fruit juices is foam layer drying. In this method, the pulp and/or juice are transformed into a stable foam through a mixer and incorporation of air or other gas, and subjected to drying with heated air until the growth of microorganisms, and chemical and/or enzymatic reactions is prevented [140]. This technique is simple, inexpensive, and fast, resulting in a porous product that is easy to rehydrate. Usually, thickening, emulsifying and stabilising agents are used, which are intended to keep the foam stable throughout the process [141]. This method’s main advantages are the need for low dehydration temperatures and a shorter drying time due to the larger surface area being exposed to air, which increases the speed of water removal [140]. Melo et al. (2013) evaluated the influence of foam thickness and drying temperature in drying the mandacaru fruit’s pulp in a layer of foam, the former being a plant belonging to the *Cactaceae*. The data obtained during the drying process concluded that the drying temperature influenced the process and foam thickness, with the fastest occurring at the smallest and highest thickness [140].

Another conservation method based on a_w_ reduction is evaporation or concentration, which may have some effects on the product. This process consists of the loss of water, being always associated with the loss of volatile compounds responsible for the flavour and aroma of the raw material. Therefore, concentrated products generally have a less intense aroma [5]. In addition, there is also a reduction in the volume of the product, which is an advantage for storage and transport. From the consumer’s point of view, concentrated products are helpful because they are easier to use, take up less space, are stored at room temperature, and can be consumed in the desired portion, while the remainder can be safely stored [6].

Freezing is a very efficient conservation technology as it allows for preserving food’s colour, aroma, texture, and nutritional and functional properties [6]. This method combines the effects of low temperature with a decrease in the water reactivity due to the formation of ice crystals. Therefore, microorganisms cannot grow, chemical reactions are reduced, and cellular metabolic reactions are delayed [5]. The PP pulp can be frozen in freezer chambers at a temperature of −30 °C and stored at −18 °C [7]. The faster the product freezes, the smaller the ice crystals that form and the better the quality of the final product. The tiny ice crystals do not damage the product’s structure. At the agro-industrial level, cold air tunnels are used (temperature of −40 °C) or the fruit is sprayed with liquid nitrogen (temperature −196 °C) [5]. The packaging material for this type of product must offer protection against oxidation, moisture loss, and changes in sensory characteristics [7]. The disadvantage of freezing is that it is an expensive technology that requires developing and maintaining a cold chain from production to consumption to guarantee the quality and safety of frozen products. Frozen pulps can be sold directly to the final consumer or manufacturers as a constituent of flavoured drinks, yoghurts, desserts, ice creams, cakes, pastries, or confectionery [6,13].

Lyophilisation is an alternative drying process for foodstuffs such as onions, apples, bananas, ginger, or pineapples. This technique consists of removing water by sublimation under reduced pressure conditions. In this way, the driving force for water removal is the vapour pressure difference between the ice and the surrounding environment. The water in the foodstuff passes from the solid phase directly to the gas without passing through the liquid phase [142,143]. Thus, lyophilisation is carried out to convert the ice into vapour without entering the liquid phase. The first step is to freeze the foodstuff to be freeze-dried at a temperature below 0 °C. Subsequently, the next step is the primary drying phase, with the sublimation of ice on the surface of the product. At this stage, the required temperature is around −10 °C, and the absolute pressure is approximately two mmHg (2.6 mBar). The last step is secondary drying, which corresponds to removing adsorbed water through evaporation. For this, applying a high-pressure gradient and increasing the temperature is necessary, but without causing damage to the product [144].

Lyophilisation can be carried out under vacuum or atmospheric pressure. The most used is the first because it allows for a final product of better quality. However, lyophilisation under atmospheric pressure has a lower energy consumption and a slightly shorter drying time [143]. Despite the high costs associated with this drying method, it becomes advantageous because it avoids the degradation of the final product due to thermal decomposition, oxidation, or enzymatic reactions [145]. On the other hand, as the lyophilisation is carried out at low temperatures, there are no changes in colour, aroma, and most of the nutritional composition of the foodstuff [143].

According to various studies, Hamad et al. (2022) summarised the physical characteristics (colour, taste/odour, and thermal properties) and the chemical characteristics (moisture content, total phenolic compound, and anthocyanin content) that changed during the spray drying (SD) and freeze drying (FD) of berry fruits. They concluded that juice taste and colour of berry powders that FD produced were better than SD, and this technique better preserved the nutritional value and bioactive compounds. On other hand, the morphology of the berry powders that underwent SD was better than the result with FD, and the losses of phenolic compounds and anthocyanin content of SD berries were much lower than FD [146].

## 6. Transformation Methods of Prickly Pear

### 6.1. Juices

One of the most common technologies for preserving fruits is the production of juices. As the PP pulp presents a variety of colours, the juices obtained become even more appealing [7]. Fresh juices should be consumed as soon as possible, preferably after their production. For a longer useful life, the juices should undergo a heat treatment to avoid pathogenic microorganism appearance without affecting the final product’s taste and appearance [5]. Generally, a high-temperature and a short-term duration heat treatment is chosen (HTST—high-temperature short time) so that the product undergoes a shorter deterioration. Subsequently, the juice must pass quickly through a system of cooling, at about 20 °C, for 30 min, to prevent overheating, which causes organoleptic and nutritional changes in the final product. In cases where the juice has been pasteurised, it must be stored at refrigeration temperature (8 °C to 10 °C), and if it has been sterilised it must be stored at room temperature (28 °C to 30 °C) [7].

The stability of juices depends not only on the raw material but also on the processing, packaging, and storage conditions. These factors can cause microbiological, enzymatic, chemical, and physical changes that damage the sensory and nutritional characteristics of the juice. Consumers demand fluids with minimal processing and no added sugar, resembling the original fruit [130]. Thus, the transformation of the PP into juices must be well established to satisfy the growing demands of consumers and encourage the consumption of this fruit [6].

The parameters that change the most during PP juice processing are the water content and the viscosity. Acidity, pigments, and aromatic compounds are the most important parameters in PP juice processing [6]. Considering some studies on PP juices, the purple ecotype seems to be the most promising in transforming the fruit into juice due to the stability of betalains. The green PP is the most challenging for juice production due to the presence of chlorophyll and its instability when subject to heat treatments, which cause changes in colour and flavour. The orange PP appears to be a good alternative for juice production but requires more studies [115].

El-Samahy et al. (2007) performed a study with orange–yellow PP juices. After obtaining the pulp, it was mixed with a sugar solution at 15 °Brix and pH 5 in a 1:1 ratio. The prepared juice was divided into three parts: the first underwent direct pasteurisation at 95 °C; the second was treated with 100 mg/L of sodium benzoate and then pasteurised at 95 °C; and the third underwent direct sterilisation at 121 °C. Subsequently, the chemical, microbiological, and sensorial characteristics of the obtained juices were evaluated during storage at room temperature (28 °C) and refrigerated temperature (8 °C) for 6 months. All juices produced were microbiologically stable during the storage period, and pasteurised juices (with or without sodium benzoate), mainly stored at refrigeration temperature, showed the best organoleptic results [139].

Kgatla et al. (2010) investigated the effects of PP juice processing and preservation on its organoleptic attributes (colour, flavour, aroma, astringency, visual appearance, and general acceptability). The PP pulp was treated with pectinase, and the treatments applied to obtaining the juice were the addition of sugar, acidification, heat treatment, refrigeration, freezing, and thawing. The reddish-purple colour of the PP remained stable for all processing and conservation. The untreated juice tasted bitter, with a high astringency, while the treated juice was significantly sweeter. The authors observed a significant difference in the acceptability of untreated and treated juices: the juries rejected the first, and the second had a good sensorial acceptance. With this study, the researchers concluded that the processing and conservation of PP juice had the positive attributes of the respective organoleptic properties [130].

Sometimes, when the heat treatments are long, the final product will likely have an unpleasant taste and/or undesirable aroma. In this way, one can resort to mixing it with other fruit juices to improve the quality of the final product [101]. Sáenz and Sepúlveda (1999) tested different formulations by mixing purple PP juice with pineapple juice, citric acid, water, and sugar. The dilution of PP juice proved to be an advantage in minimising its viscosity, and the addition of citric acid and pineapple juice lowered the pH, which reduced the risk of microorganism growth [115].

Since PP juices have a short shelf life, Ferreira et al. (2022) conducted a study where they applied thermal (TP) and high-pressure (HPP) pasteurisation to OFI juices from three different cultivars from Idanha a Nova (Portugal). They also evaluated the impact of these methods on microbial safety, physicochemical properties, and nutritional content over storage at 4 °C. They concluded that TP at 71.1 °C for 30 s increased the shelf life by 22 days, and HPP at 500 MPa for 10 min increased the shelf life by 52 days relative to microbial growth and to the preservation of physicochemical properties. Applying these pasteurisation methods retarded the physicochemical changes, namely in titratable acidity, °Brix, browning, polyphenolic content, and antioxidant activity of the juices [147].

Other types of products can be made from the PP juice, such as concentrates or nectars, prepared by direct concentration or by adding sugar. In the case of nectars, the production steps are identical for obtaining juice, incorporating sucrose or corn syrup, and sometimes some additives, such as carboxymethylcellulose, to give body to the final product [7]. The main advantage of these products is having a lower a_w_ in relation to juices, which leads to an increase of the product shelf life due to lower chemical reactivity and greater protection against the development of microorganisms [84].

### 6.2. Fermented Products: Beverages and Vinegar

Artisanal alcoholic beverages can be obtained by juice fermentation, such as “colonche” (4 to 6% alcohol), wine (reaching about 11% alcohol), and brandy (reaching up to 56% alcohol). Other types of products that have been emerging are vinegar and liqueurs based on PP or based on mixtures with other fruits [7].

Lee et al. (2000) fermented various mixtures of PP juice (PPJ) and grape juice (GJ) to produce alcohol. Fermentation was carried out using *Saccharomyces cerevisiae* with the addition of sulphur dioxide (SO_2_), sodium sulphite (Na_2_SO_3_), and tartaric acid (C_4_H_6_O_6_) to adjust the acidity. The fermentation of the PPJ could have been more successful, but it was progressing with the addition of the GJ. The mixture of PPJ (25%)/GJ (50%) was fermented at 30 °C, for 7 days, and presented an alcohol content of 9.2% (*m*/*V*). The mixture of PPJ (70%)/GJ (30%) produced an alcoholic beverage with 6.9% (*m*/*V*) of alcohol, and alcoholic fermentation of the mixture of PPJ (50%)/GJ (50%) was carried out at 22 °C for 6 days [148].

Turker et al. (2001) used PPJ as a raw material for fermentation and *Saccharomyces cerevisiae* as a fermentation microorganism. The fermentation process allowed the conversion of 95.54% of the fermentable sugar, with an ethanol yield of 55.3 mL/L. According to the results obtained, the authors concluded that the fermentation process did not affect the thermostability of PP betalains [149].

Pérez et al. (1999) prepared vinegar from orange PP. To carry out the alcoholic fermentation at 13.5 °GL, they used *Acetobacter pasteurianus*, and for the fermentation of PPJ with added sugar at 22 °Brix they used *Acetobacter xylinum*. The vinegar produced in both cases had a clean, bright, intense amber–yellow colour, and an acidic, fresh, and intense aroma. Different kinds of vinegar can be developed depending on the wide range of colours of the PP [5].

Es-sbata et al. (2022) produced PP vinegar using two types of acetification processes (surface and submerged culture), at different temperatures (30, 37, and 40 °C), by using two different species of thermotolerant acetic acid bacteria (*Acetobacter malorum* and *Gluconobacter oxydans*). Then, 15 polyphenols and 70 volatile compounds were identified and quantified in the PP vinegar samples produced by both acetification processes, but the phenolic content from surface culture acetification was higher, and the submerged culture was a faster and more efficient acetification method because of the higher concentration of acetic acid in the PP vinegar. With this study, they verified that PP vinegar can be successfully produced at higher temperatures than usual by employing thermotolerant bacteria and that the type of acetification method significantly affects the final quality of the vinegar produced [150].

### 6.3. Sweets, Jams, and Jellies

Another technological alternative is sweets, jams, and jellies, which are obtained by boiling the PP pulp with ingredients such as sugar, citric acid (implements taste and microbiological stability), pectin (responsible for viscosity due to its water holding capacity), and preservatives such as sodium sorbate or potassium benzoate (they preserve the quality of the product after opening the package), followed by concentration by evaporation to ensure a certain degree of gelation [6,7].

Sawaya et al. (1983) reported the manufacture of PP jam. In this work, they studied the effects of the sugar:pulp ratio, acidifying agents (various types and amounts), the pectin dosage, aroma, the date:PP ratio, and the bleaching effect of fruit on the quality of the compote. The best sensory results were obtained with the following recipe: pulp:sugar ratio of 60:40, adding 1.25% pectin, citric acid, or a combination of citric and tartaric acids (1:1), with a 20% date ratio. Regarding the added flavours, the favourites were clove, grapefruit, orange, and almond [73].

Saenz et al. (1997) studied green PP pulp gels to which sugar and carrageenan were added. Although a significant colour change was observed because of the chlorophyll phaeophytinization due to decreased pH, the product maintained its physicochemical and sensory properties for more than 14 days when refrigerated between 4 and 6 °C. They also concluded that refrigeration could be avoided if they increased the concentration of sugar, which is a common commercial practice [6].

Razafindratovo et al. (2022) carried out a study focused on the nutritional characterisation of PP pulp and processed products (jam and syrup). They concluded that the jam and syrup made from the PP pulp were products with interesting nutritional, organoleptic, and physicochemical characteristics, which complied with manufacturing standards [151].

Abu-shama et al. (2022) studied jelly candies prepared by using six formulas of PP juice and peels (yellow and red cultivars). The obtained results showed that the jelly candies produced were an important source of total polyphenols, flavonoids, carotenoids, and betalains. The jelly candies produced could be stored for more than four weeks during cold storage and had an acceptable quality to consumers: the sensorial evaluation showed that red peels, red juice, and yellow juice jelly candies were the most accepted by panellists [152].

### 6.4. Oil

The PP seed oil has several physicochemical and nutritional supplements, making it like other edible vegetable oils, such as corn or grape [84]. After extracting the seeds from the fruit, they are cleaned and dried before starting the cold extraction process [7].

On the one hand, this oil can be used as a fat substitute in confectionery, and the by-product obtained from its extraction can be used for animal feed. On the other hand, PP seeds produce a low of oil yield due to the number of seeds obtained, and are only profitable if the extraction is associated with processing, whether of pulps, juices, or jams [5,7,84].

Ammar et al. (2017) carried out a study focusing on evaluating the quality and oxidative stability of olive oil (very important in Mediterranean cuisine) with added OFI flowers. The quality, fatty acids profile, total phenol contents, and thermal properties of the olive oils enriched with OFI flowers were studied. The oxidative stability of olive oils was improved, mainly in olive oil enriched with 5% OFI flowers, after 15 and 30 days of storage at 60 °C. The olive oil was also nutritionally enriched due to the increase in its phenols content [153].

### 6.5. Flour

Another product from various OFI processing techniques is flour from cladodes, seeds, and PP peel. The OFI flour can be used to make cookies, puddings, cereals, tortillas, and various snacks, or to produce food supplements in the form of capsules or tablets, allowing increased daily dietary fibre intake [5].

OFI flour can be obtained by dehydrating and grinding cladodes, previously degreased, washed and cut, or by grinding the seeds and/or peel, after which it passes through two sieves of different granulometry to separate the fibres [6,7]. The result is simple and inexpensive flour that can be mixed with other flour to improve its taste, smell, colour, and texture [5].

Ayadi et al. (2009) studied the fortification of wheat flour with OFI cladode flour for obtaining cakes. As a source of fibre, cladodes increase the flour’s water absorption capacity and, consequently, the cake dough’s properties. The results indicated that adding cladode flour increased the tenacity and decreased the elasticity of the cake dough. In terms of colour, the cakes containing cladode flour became greener inside and darker outside. The best sensory results were with the cakes with 5% of cladodes flour [154].

In the study of Msaddak et al. (2017), the effects of OFI cladodes powder substitution of wheat flour on dough’s rheological, physical, and antioxidant properties and sensory bread characteristics were analysed. It was found that, in wheat bread formulation, a substitution of up to 5% of wheat flour by cladodes powder was possible without changing the physical and sensory properties. This change improved the total phenolic content and the bread’s antioxidant potential without negatively affecting its sensory acceptability [155].

### 6.6. Snacks

Snacks are defined as light meals consumed between regular meals and may include a wide range of products, such as cookies, cereal bars, yoghurts, and even ice cream [156]. Within this category, cookies are the most popular bakery products consumed worldwide due to their various characteristics: ready-to-eat, reasonable cost, high nutritional value, long shelf life, and availability in different colours, shapes, and flavours [157].

The production of cookies of acceptable quality is based on the selection of flour and the appropriate processing steps, such as mixing, aeration, fermentation, cooking, cooling, and packaging [158]. The main constituents of cookies are flour, wheat, sucrose, and fat, which make them a very dense food. Due to their low moisture content, cookies can serve as a source of various nutrients. There is a tendency to produce functional cookies made from wheat flour, with a good source of calories, different nutrients, and other flours containing active compounds [159,160].

Abou-Zaid et al. (2022) determined the chemical characteristics of fresh juice and peel of both OFI (yellow cultivar) and *Opuntia littoralis* (red cultivar), and used them in cookies production and evaluated the quality of the cookies. The results showed that fresh PP juices and peels could be used to produce healthy and organoleptically appealing cookies. The cookies produced with juices had a higher moisture content than those produced with peels, and those produced with peels had the highest crude fibre contents and the highest weight and hardness [30].

To take advantage of the by-products of fruits and vegetables and recover the individual dietary fibres, bioactive compounds, and antioxidants, Elhassaneen et al. (2016) produced snacks with flour from PP and potato peels. The peels were dehydrated under vacuum, at 70 °C, for 3 h to obtain flours with 7% humidity. Subsequently, they incorporated the flour into the cookie dough at levels of 5%. Biscuits enriched with the by-products studied showed higher fibre contents, total dietary intakes, carotenoids, and total phenolic compounds in comparison with control biscuits. In this way, the authors concluded that, by incorporating the flours from the PP and potato peels, it was possible to improve the nutritional and functional quality of the cookies without affecting their sensory characteristics [156].

Mahloko et al. (2019) developed and evaluated cookies obtained by mixing flour from PP and banana peels in another study with a similar objective. The flour was dehydrated at 60 °C overnight and incorporated into the wheat flour for the biscuit production. The results obtained indicated that the cookies enriched with the flours from PP and banana by-products contained higher levels of fibre, total phenolics, and flavonoids than the control. In general, they concluded that incorporating flour from PP and banana peels improved cookies’ functional properties, colour, and antioxidant activity [160].

El Samahy et al. (2007) produced another type of value-added snack based on PP pulp. In this study, they concentrated the yellow–orange and red PP pulps to 40 °Brix and added them to the rice flour. Different formulations were tested, varying the relationship between rice flour and PP pulp concentrate. The products that obtained the best functional, nutritional, and sensory characteristics contained 5% and 10% PP pulp concentrate [13,161].

### 6.7. Dairy Products

Another way to use PP is to obtain dairy products. One of these products is PP “cheese”, obtained from concentrated juice, which is boiled until reaching the desired consistency. After cooling, the product is a compact, malleable mass with a high sugar content, like caramel. Then it is struck on a smooth, moist stone platform to allow air to enter and prevent the formation of sugar crystals. Before packaging, the dough is shaped and kept for 12 to 15 h. This product must be slightly heated and can be enriched with vanilla, pine nuts, hazelnuts, walnuts, shredded coconut, raisins, or almonds [7].

Milk permeate is a by-product of milk ultrafiltration, containing 80% of the lactose starting point of treated milk, and is an excellent source of vitamins and minerals [162]. To reduce the environmental pollution inherent in the elimination of permeate, dairy industries try to arrange new destinations for the permeate, such as the preparation of fermented milk or the production of chocolate milk [163,164]. Bearing this perspective in mind, Jambi et al. (2017) looked at producing PP-based beverages by mixing different concentrations of PP pulp and permeate. They studied the different mixtures’ physicochemical, microbiological, and organoleptic properties after production and storage at 4 °C, over 7, 14, and 21 days. The obtained results indicated that the acidity and the levels of phenolic compounds and ascorbic acid increased as the PP pulp concentration increased in the beverages, unlike the pH, which decreased. Over storage time, phenolic compounds and ascorbic acid content decreased in all mixtures. The lactose content of the drinks also reduced over time of storage. The beverage that contained 30% of PP pulp and 70% permeate had the highest organoleptic ratings, both immediately after its preparation and after 21 days of storage at 4 °C [165].

### 6.8. Sweeteners

Based on juice technology, there is the possibility of obtaining natural sweeteners with a PP base through the treatment of enzymatic clarification, using pectinolytic enzymes with high arabinase activity. During the process, the acidity of the PP juice is corrected with citric acid to pH 4.2 to 4.5, so the enzyme works. This is followed by filtration, decolourisation with activated carbon, a new filtration, and concentration until a product with 60 to 62 °Brix is obtained. The final product has a yellowish colour and a sweetness like other commercial liquid sweeteners, and is packaged in glass or polyethylene bottles and stored at room temperature [7]. Saenz et al. (1996, 1998) were the first to develop a process for obtaining a natural liquid sweetener from PP juice. The final product, light golden yellow, had 60 °Brix (56% glucose and 44% fructose), a 1.29 g/mL density, a_w_ of 0.83, and a viscosity of 27.1 cps, like honey or marmalade [84].

### 6.9. Natural Dyes

In addition to obtaining flour, there is another type of recovery of the PP peel: extraction of its pigments, which differ depending on the ecotype, and the extraction of pectin [16,119]. The pigments that PP can offer range from chlorophyll (green colour), betacyanin (purple colour) and betaxanthin (yellow–orange colour), and they are a promising source of natural dyes. Aqueous solutions of mucilage extracted from the fruit can be used as a source of fibre, as a thickening agent for culinary purposes, and as an edible coating to protect fresh fruit [5,6,7].

Del-Valle et al. (2005) studied the mucilage extracted from PP as an edible coating to prolong the shelf life of strawberries stored at 5 °C. In addition, to investigate different methods of mucilage extraction to obtain the best coating, they also analysed the effects of the other coatings on the colour, texture, and sensory quality of strawberries. According to the results obtained, they concluded that, besides the mucilage coatings increasing the shelf life of strawberries, the latter also maintained their texture and flavour, with no deterioration after 9 days of storage [166].

PP peels are cheap, readily available, and their physicochemical properties can be used to improve food product parameters such as shelf life, sensory characteristics, and viscosity. They may also be applied as fat or sugar substitutes to stabilise food oxidative processes, and as oil and water retention capacity enhancers [3].

Chougui et al. (2015) conducted a study to evaluate the use of PP peel hydroethanolic extracts as a substitute for vitamin E, used as an antioxidant in margarine conservation. The tests indicated that the margarine prepared with three different concentrations of PP peel extract was more resistant to oxidation than margarine containing vitamin E. In addition, the physicochemical and microbiological properties of margarine were not modified. The lowest concentration of analysed extract (50 mg/kg) showed a higher margarine shelf life [167].

## 7. Case Study: Agro-Industrial Uses in Portugal and Prospects for Market

The PP is a fruit with potential agro-industrial expansion as it is a source of several nutrients and an efficient system to produce various foods, such as juices, jellies, pulps, oil, or flour [7]. The technological transformation that this fruit can undergo becomes very relevant due to the decrease in water resources, the increase in global desertification, the maximum use of resources, and how the plant adapts to arid lands and severely degraded soils, unsuitable for traditional cultures, and is important from the sustainability point of view [5]. On the other hand, the PP has characteristics of specific seasonality and rapid post-harvest deterioration, which compromises its storage and marketing, so the agro-industrial applications aim at better use of this fruit, a reduction in production losses, and a diversification in the number of new products derived from this fruit [7,42].

Portugal, namely the Alentejo and Algarve, has soil and climate conditions making it possible to obtain high-quality PP production compared with other countries [7]. In Portugal, the PP crop is expanding, with more than 800 ha cultivated between two to four years [40].

Since 2008, the PP crop has existed in an orderly fashion in Portugal under the rural development programme (ProDer). The objectives of this programme are to support investment in agriculture, boost young farmers, and help the development of small and micro-enterprises [7]. To this end, a project was started that encompasses the study of culture, the creation of a collection of national PP ecotypes, the study of physicochemical and nutritional characteristics of the fruit, and the development of innovative food technology. Subsequently, and with the increase in the planted area, associations have been created within the scope of production, marketing, and cultural dissemination around the culture of the prickly pear [7].

Portugal has adopted specific production systems to prolong the harvest season to satisfy market needs and compete with other countries’ PP exporters. Using agronomic techniques, it is possible to change the biological cycle of the OFI, such as prolonging the flowering season of the plantation, which promotes late flowering [40,42].

Bearing in mind that PP is characterised as a tropical fruit with several nutritional advantages, the introduction of national production in the domestic market is simply due to the growing interest in healthy foods and lifestyles. On the other hand, consuming this fruit and its processed products can increase its commercial and economic value at the national level [7,42,57].

The PP is for sale in some local markets and distribution chains, and its prices vary between EUR 2.99 and 14.99 /kg. This price variation is due to several factors, such as country of origin, type of packaging, size, and fruit quality [7]. As for exports, the percentage is still residual, and although no data statistics show international sales volumes, potential countries’ PP buyers are England, France, and Japan [7].

## 8. Conclusions

In summary, the physicochemical and nutritional composition of OFI is only partly known because most of the investigations were done 10–20 years ago. Hence, this needs to be validated with up-to-date methods. This is an essential issue for OFI applications and better agro-industrial use.

The PP is a source of several nutrients and an effective system to produce varied foods, which have several advantages from a nutritional, sensory, economic, and shelf-life point of view. The technological transformation that this fruit can undergo becomes very relevant due to the decrease in water resources, the increase in global desertification, and the maximum use of resources. Due to its adaptation to arid lands and severely degraded soils, which are unsuitable for traditional cultures, it is also important from a sustainability point of view. On the other hand, the PP has specific seasonality characteristics and rapid post-harvest deterioration, compromising storage and marketing. Therefore, agro-industrial applications aim to use this fruit better, reduce production losses and diversify the offer of new products derived from this fruit.

Finally, it is essential to note that about one-third of the Portuguese territory is highly susceptible to desertification. Climate change could exacerbate the effects of droughts, and accelerate soil degradation and, consequently, the desertification of the region, severely conditioning the development of extensive rural areas. OFI cultivation can contribute to revitalising these rural areas and the dynamism of local economies, combating the depopulation process that affects them. In addition to producing fruits with excellent nutritional properties, its production allows owners of uncultivated or underused land to obtain a significant and sustainable income and stimulate upstream and downstream economic activities.

## Figures and Tables

**Table 1:** Taxonomic classification of *Opuntia ficus-indica* (L.) Mill [32,33,34].

OFI Taxonomic	
Domain	*Eukaryota*
Kingdom	*Plantae*
Subkingdom	*Embryophyta*
Phylum	*Spermatophyta*
Division	*Magnoliophyta*
Class	*Magnoliopsida*
Subclass	*Caryophyllidae*
Order	*Caryophyllales*
Suborder	*Cactineae*
Family	*Cactaceae*
Subfamily	*Opuntioideae*
Tribe	*Opuntieae*
Genus	*Opuntia*
Subgenus	*Plantyopuntia*
Species	*Opuntia ficus-indica* (L.) Mill

## Data Availability

Data sharing is not applicable to this article.
